# Discovery of aminopyridine-containing spiro derivatives as EGFR mutations inhibitors

**DOI:** 10.1080/14756366.2019.1634704

**Published:** 2019-07-09

**Authors:** Lianbao Ye, Tao Zhao, Wenjun Du, Anhu Li, Wei Gao, Jingrong Li, Ling Wang, Weiqiang Chen

**Affiliations:** a School of Pharmacy, Guangdong Pharmaceutical University, Guangzhou, China;; b Esa Biotech Co., LTD., Guangzhou, China;; c School of Basic Courses, Guangdong Pharmaceutical University, Guangzhou, China;; d Joint International Research Laboratory of Synthetic Biology and Medicine, Guangdong Provincial Engineering and Technology Research Center of Biopharmaceuticals, School of Biology and Biological Engineering, South China University of Technology, Guangzhou, China

**Keywords:** EGFR/HER2 inhibitors, aminopyridine, spiro derivatives, EGFR mutations, molecular docking

## Abstract

Neratinib is an oral pan HER inhibitor, that irreversibly inhibits EGFR and HER2 and was proven to be effective against multiple EGFR mutations. In previous study, we reported spiro [indoline-3, 4′-piperidine]-2-ones as anticancer agents. In this study, we designed aminopyridine-containing spiro [indoline-3,4′-piperidine] derivatives **A1-A4** using Neratinib and spiro [indoline-3, 4′-piperidine]-2-one compound patented as lead structure, then replaced piperidine with cyclopropane to obtain **B1-B7** and replaced indoline with benzmorpholine to get **C1-C4** and **D1-D2**. We synthesized these compounds and evaluated their residual activities under 0.5 M drug concentration on EGFR and ERBB2. Most of compounds showed stronger inhibition on EGFR-wt and ERBB2, in which **A1-A4** showed excellent inhibitory activity with inhibition percentage on EGFR-wt kinase of 7%, 6%, 19%, 27%, respectively and 9%, 5%, 12%, 34% on ERBB2 kinase compared with 2% and 6% of Neratinib.

## 1. Introduction

Overexpression of epidermal growth factor receptor (EGFR or HER1) and human epidermal growth factor receptor (HER2) is frequently found in different solid tumors. Coexpression of EGFR and HER2 has been reported in different tumors. They are also accompanied with postoperative adverse, radiotherapy, and chemotherapy resistance^1–3^. Therefore, it is more effective to dual target EGFR/HER2 rather than just EGFR inhibition^4^. Targeting of EGFR and HER2 is a proven anti-cancer strategy^5^. Both kinases have been an attractive therapeutic targets for cancer therapy. A variety of ATP-competitive EGFR/HER2 RTK dual inhibitors related to different scaffolds have been reported and many of them are currently in market or clinical trials for the treatment of cancer^6–8^.

Neratinib (HKI-272) is a highly selective inhibitor of HER2 and EGFR that leads to reduced phosphorylation and activation of downstream signaling pathways^9^ ^,^ ^10^. Neratinib was proven to be effective against multiple EGFR mutations including T790 mutation, L858R mutation and T790/L858. In complex with Neratinib, the EGFR kinase adopts an inactive conformation in which the regulatory C-helix is displaced from its active position. The enlarged hydrophobic pocket created by the outward rotation of the C-helix appears to be required to accommodate the bulky aniline substituent found in Neratinib, which contain additional aromatic groups appended to the 2-pyridinyl group and binds the inactive conformation of the kinase^11^ ^,^ ^12^. Neratinib is currently being tested in a number of clinical trials for its safety and efficacy in lung cancer, and colorectal, bladder, and breast cancers^13–15^.

**Figure 1.**
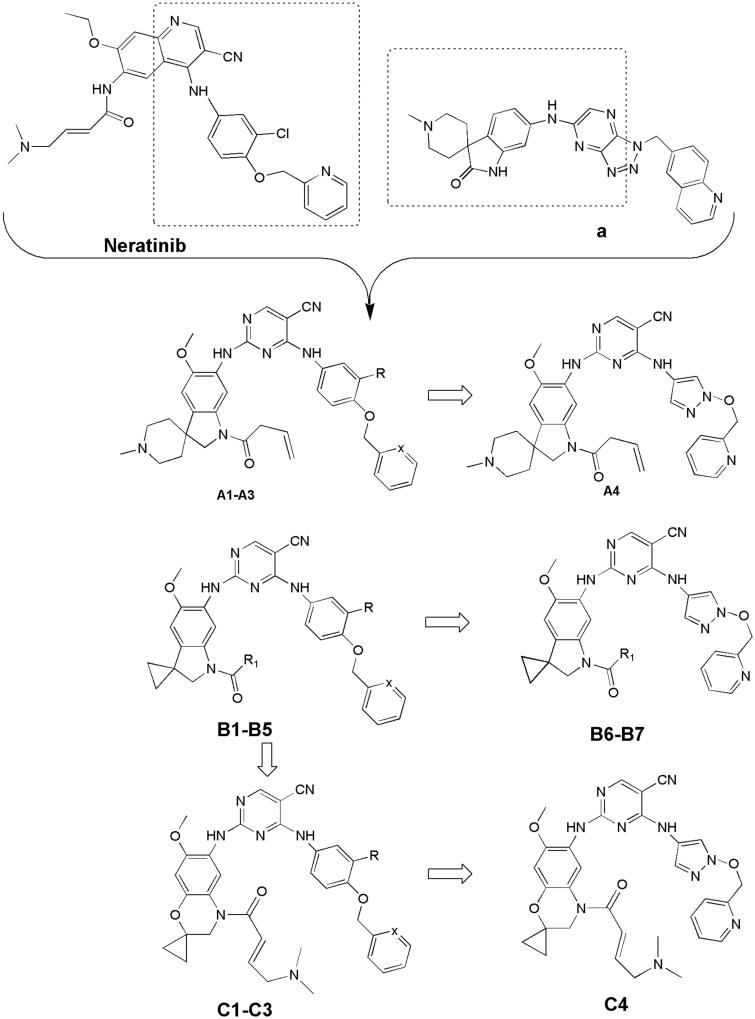
Design of target compounds.

In previous study, we reported spiro [indoline-3, 4′-piperidine]-2-ones as anticancer agents and several compounds showed stronger inhibition on tyrosine kinase^16^. In this study, we designed aminopyridine-containing spiro [indoline-3, 4′-piperidine] derivatives **A1-A4** using neratinib and spiro [indoline-3, 4′-piperidine]-2-one compound (**a**) patented as lead structure, then replaced piperidine with cyclopropane to obtained **B1-B7** and replaced indoline with benzmorpholine to get **C1-C4** (Figure 1 and Tables 1–3). We synthesized these compounds and evaluated their residual activities under 0.5 M drug concentration on EGFR-wt and ERBB2. Then, we performed head to head comparative trial with neratinib to investigate inhibitory effects of compounds (**A1-A2)** with the best activity on EGFR-wt, HER2 and EGFR mutations. Molecular docking was adopted for all the synthesized compounds to confirm their mechanism of action.

**Table 1.**
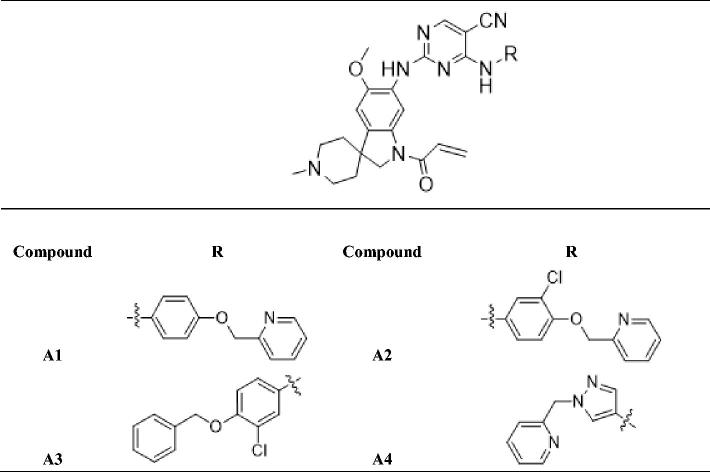
Novel aminopyridine-containing spiro derivatives **A1-A4**.

**Table 2.**
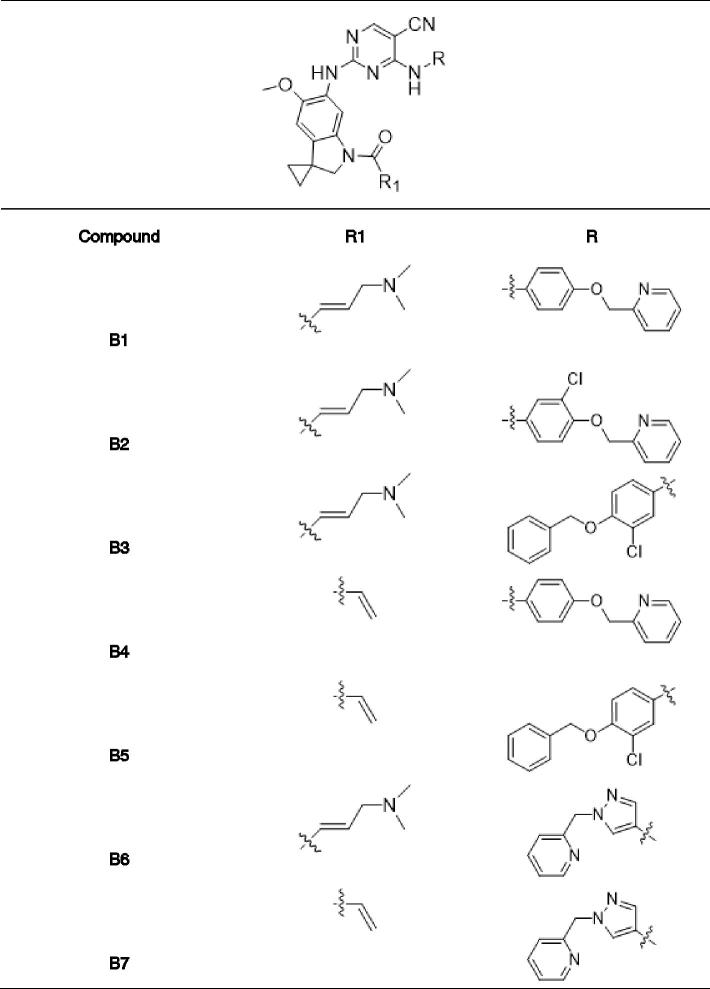
Novel aminopyridine-containing spiro derivatives **B1-B7**.

**Table 3.**
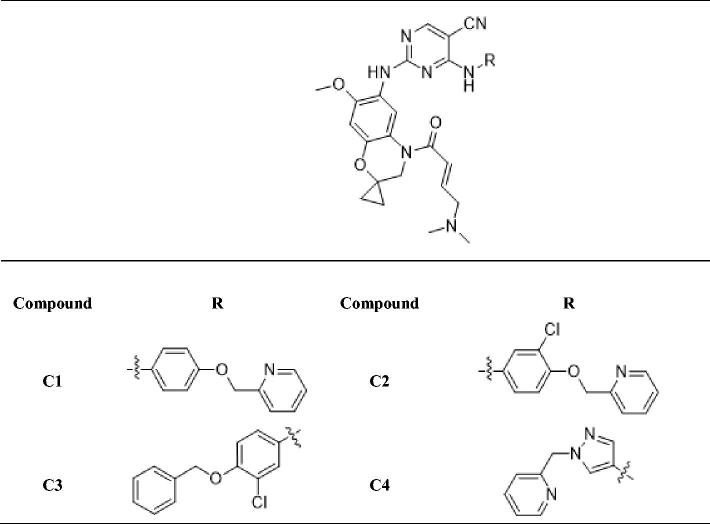
Novel aminopyridine-containing spiro derivatives **C1-C4**.

## 2. Results and discussions

### 2.1. Chemistry

**Figure 2.**
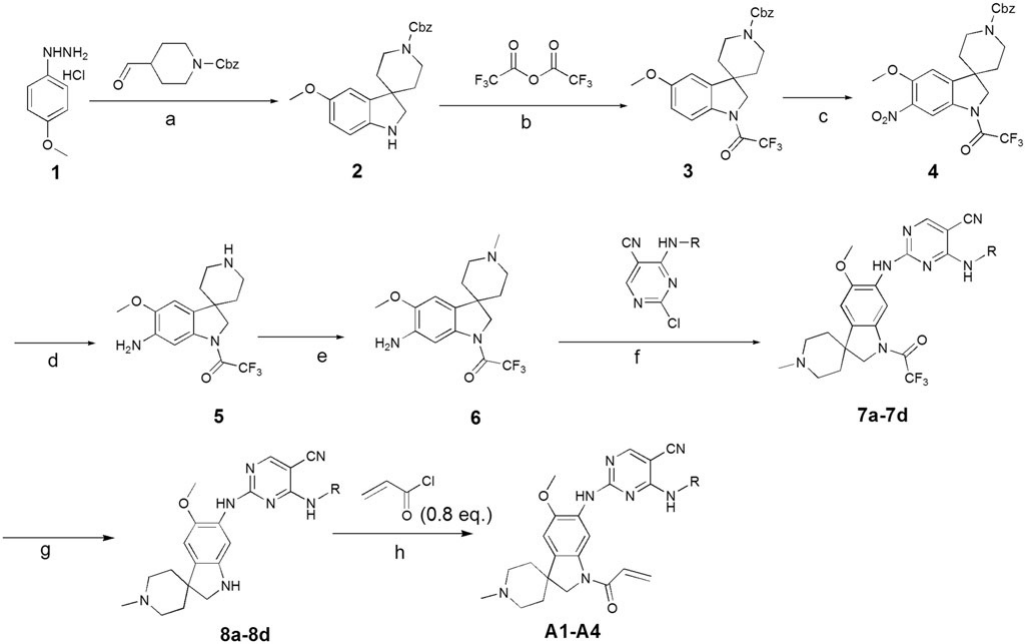
Synthesis of A1-A4, Reagents and conditions: (a) (i) TFA, Toluene, rt, (ii) NaBH_4_, MeOH, rt; (b) TEA, DCM, 0 °C; (c) Cu(NO_3_)_2_.3H_2_O, Ac_2_O; (d)Pd/C, H_2_, rt; (e) MeI, TEA, THF, rt; (f) PTSA, 2- propanol, dioxane, 80 °C; (g) K_2_CO_3_, MeOH, rt; (h) TEA, DCM, –20 °C.

**Figure 3.**
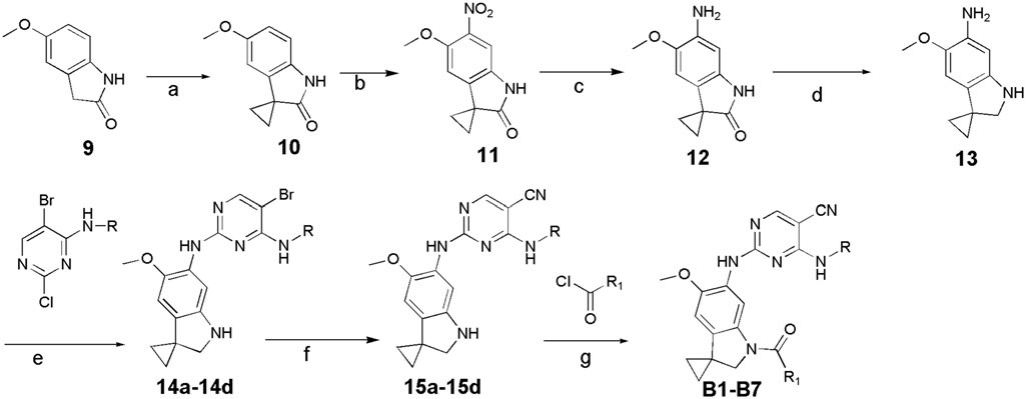
Synthesis of B1-B7, Reagents and conditions:(a) (i) iPr_2_NH, nBuLi, THF, –60 °C, (ii) BrCH_2_CH_2_Br, 60 °C; (b) HNO_3_, Ac_2_O, DCM, rt; (c) Fe, NH_4_Cl, MeOH, H_2_O; (d) LiAlH_4_, THF, 70 °C; (e) pTSA, Dioxane,100 °C, 2 h; (f) Zn(CN)_2_, DMF, H_2_O, 120 °C(MW)/90 min; (g) TEA, DCM, 0 °C, 2 h.

**Figure 4.**
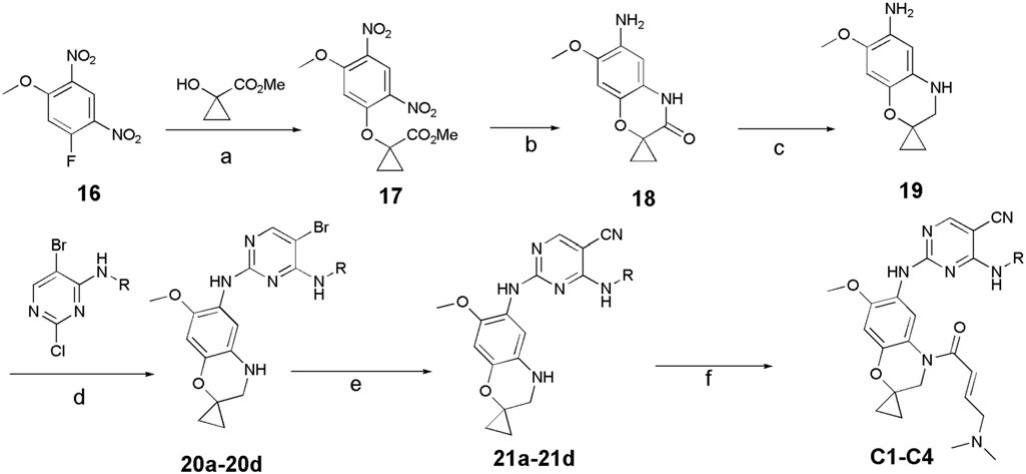
Synthesis of C1-C4, Reagents and conditions: (a) Cs_2_CO_3_, MeCN, rt, 2 h; (b) Fe/NH_4_Cl, MeOH, H_2_O,70 °C,12h; (c) LAH, THF, 70 °C, 2 h; (d) pTSA, Dioxane, 100 °C, 2 h; (e) Zn(CN)_2_, Pd_2_(dba)_3_.CHCl_3_/dppf, DMF, H_2_O, 120 °C(MW), 1.5 h; (f) (E)-4- (dimethylamino)but-2-enoyl chloride, TEA, 40 °C, 2 h.

In previous study, we reported spiro [indoline-3, 4′-piperidine]-2-ones as anticancer agents. In this study, we designed aminopyridine-containing spiro [indoline-3,4′-piperidine] derivatives **A1-A4** using Neratinib and spiro[indoline-3, 4′-piperidine]-2-one compound patented as lead structure. The synthetic route of compounds **A1-A4** was shown in Figure 2. The 1-{6-amino-5-methoxy-1′-methyl-1,2-dihydrospiro[indole-3,4′-piperidine]-1-yl}-2,2,2-trifluoroethan-1-one **(6)** reacted with 2-chloro-4-aminopyrimidine-5-carbonitriles with different substituents on amino nitrogen to obtain **7a-7d** using p-toluenesulfonic acid as catalyst with the yield of about 70% and compounds 7a-7d were hydrolyzed to get **8a-8d**, which were transferred to target compounds **A1-A4** by acylation of acryloyl chloride. The compound **6** was prepared using (4-methoxyphenyl)hydrazine hydrochloride as starting material through condensation reaction, acylation reaction, nitration, reduction reaction and methylation. The piperidine of **A1-A4** was replaced by cyclopropane to obtain **B1-B7**, which were synthesized according to Figure 3. Compounds **B1-B7** were obtained by acylation of **15a-15d**, which was synthesized by the reaction of **14a-14d** and zincdicarbonitrile using tris(dibenzylideneacetone)dipalladium and 1,1′-ferrocenebis(diphenylphosphine) as catalyst and N, N-dimethylformamide and water as solvents. Compounds 14a-14d were prepared by the reaction of the spiro compound 5′-methoxy-1′, 2′-dihydrospiro[cyclopropane-1,3′-indole]-6′-amine (**13**) and 2-chloro-4-aminopyrimidine-5-carbonitriles with different substituents on amino nitrogen. The compound **13** was synthesized using 5-methoxy-2, 3-dihydro-1H-indol-2-one (**9**) as starting material through alkylation, nitration, reduction reaction. The indoline structure of **A1-A4** and **B1-B7** was replaced with benzmorpholine to obtain **C1-C4.** The synthetic route of compounds **C1-C4** was shown in Figure 4. Firstly, spiral intermediates 7-methoxy-3,4-dihydrospiro[1,4-benzoxazine-2,1′-cyclopropane]-6-amine **(19)** and 7-methoxy-1′-methyl-3,4-dihydrospiro[1,4-benzoxazine-2,4′-piperidine]-6-amine **(26)** were prepared using 1-fluoro-5-methoxy-2,4-dinitrobenzene as starting material. Then, the compound **19** was used to synthesize **C1-C4** by taking a similar approach to prepare **A1-A4** and **B1-B7**, respectively.

### 2.2. Biological evaluation

#### 2.2.1. EGFR-wt/HER2 protein kinase assay

The inhibitory profile of 17 compounds was determined using two protein kinases EGFR-wt and HER2. All values are residual activities (% of control activity) were shown in Table 4. The testing of 17 compounds in singlicate at one concentration (5 × 1 0^−7 ^M) with two protein kinases showed a differential inhibitory profile. Residual activity of compounds as followed: Neratinib and **A1-A3 **≤** **20%, A4 > 20% and ≤60%, C1,C3, B5 > 60% and ≤80%, the other compounds >80%. As can be seen from the results, compounds **A1-A4** were lucky to show stronger inhibition on EGFR-wt and HER2 with inhibition percentage on EGFR-wt kinase of 7%, 6%, 19%, 27%, respectively and 9%, 5%, 12%, 34% on ERBB2 kinase, in which **A1** and **A2** was pretty much to neratinib with residual activities of 2% and 6%. Since **A1** and **A2** had superior activity on EGFR-wt, they will almost certainly be effect on various types of EGFR mutations, and was the strong possibility that **A1** and **A2** could therapy both HER2-positive breast cancer and lung cancer-resistance to the first and the second line EGFR inhibitors compared to neratinib. The compounds revealed that spiro[indoline-3, 4′-piperidine] could increase the activity. Further research works are currently under investigation and will be reported in due course.

**Table 4.** Residual activities under 0.5 drug concentration of compounds on EGFR-wt and HER2.

	Residual activity under 0.5 μM drug concentration (%)	
Compound	EGF-R wt	HER2
Neratinib	2	6
A1	7	9
A2	6	5
A3	19	12
A4	27	34
B1	92	103
B2	97	100
B3	95	102
B4	87	99
B5	68	102
B6	94	113
B7	89	135
C1	86	100
C2	76	108
C3	79	111
C4	85	100
DMSO	100	113
DMSO	88	123
DMSO	105	110

#### 2.2.2. EGFR wt, HER2, and seven EGFR mutants protein kinase assay

Based on good inhibitions of **A1-A2** on EGFR-wt and HER2, we performed head to head comparative trial with neratinib to investigate inhibition of **A1-A2** on EGFR-wt, HER2 and seven EGFR mutations (EGF-R d747-749/A750P, EGF-R d752-759, EGF-R G719S, EGF-R L858R, EGF-R L861Q, EGF-R T790M,EGF-R T790M/L858R). The IC_50_ values for three compounds in 9 protein kinase assays are shown in Table 5. The corresponding IC_50_ curves of three compounds in 9 protein kinase assays are shown in Supporting Information. The results confirmed that **A1** and **A2** were dual EGFR/HER2 inhibitors, biochemical activities on ERBB2 were about the same to neratinib with the IC_50_ values of 0.18 μM, 0.26 μM, and 0.31 μM, respectively, biochemical activities on EGFR wt were not more than that of neratinib but within five times. However, **A1** and **A2** had stronger inhibitory effect on various EGFR mutants, especially on T790M and L858R that were main mutants resistant to EGFR inhibitors in lung cancer. The inhibitory activities of **A1** and **A2** on EGFR mutants containing both T790M and L858R were 31 times stronger with IC_50_ values of 0.09 μM and 0.08 μM than neratinib of 2.5 μM. These results showed that **A1** and **A2**could be developed as potential inhibitors to therapy breast cancer and drug-resistant lung cancer.

**Table 5.** Biochemical IC_50_ values of compounds against EGFR wt, HER2, and seven EGFR mutants protein kinase.

Protein kinase	IC_50_ values μM		
	Neratinib	A1	A2
EGF-R d747-749/A750P	0.67	0.053	0.054
EGF-R d752-759	0.17	0.21	0.2
EGF-R G719S	0.019	0.2	0.24
EGF-R L858R	0.08	0.086	0.081
EGF-R L861Q	0.017	0.076	0.081
EGF-R T790M	0.17	0.089	0.13
EGF-R T790M/L858R	2.5	0.09	0.08
EGF-R wt	0.014	0.061	0.059
HER2	0.18	0.26	0.31

### 2.3. Docking study

The main structural difference between the active and inactive states is a structural change in the TKD activation loop and movement of the N-lobe helix, both located near the adenosine triphosphate (ATP) binding site. Unlike the active and inactive conformations present in most RTKs and EGFR, in HER2 the conformation as experimentally identified is in the middle of these active–inactive conformations, and has been named the “active-like conformation”, due to the orientation of the helix-αC-ac, the DFG-in and the unformed secondary structure of the activation loop^17^ ^,^ ^18^.Structural analysis showed that the drugs impact differently the conformational space of active and inactive EGFR and energetic analysis pointed out that some ligands have better affinity for the inactive EGFR than the active EGFR state^19–21^.

The reports show that in complex with neratinib, the EGFR kinase adopts an inactive conformation in which the regulatory C-helix is displaced from its active position and the enlarged hydrophobic pocket created by the outward rotation of the C-helix appears to be required to accommodate the bulky aniline substituent found in neratinib, neratinib contain additional aromatic groups appended 2-pyridinyl group and binds the inactive conformation of the kinase, the quinoline core of neratinib forms a single hydrogen bond with the hinge region of the kinase. The 2-pyridinyl group of neratinib is surrounded by hydrophobic residues in the expanded pocket, including Met-766 in the C-helix, Phe-856, and Met-790, the mutant gatekeeper residue, the nitrile substituent of neratinib also approaches the gatekeeper residue^11^ ^,^ ^12^.

**Figure 5.**
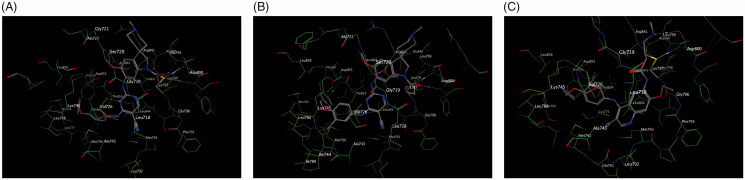
Docking of compounds the active site of 2JIV: (A) Docking of A1 to the active site of 2JIV. (B) Docking of A2 to the active site of 2JIV. (C) Docking of Neratinib to the active site of EGFR.

**Figure 6.**
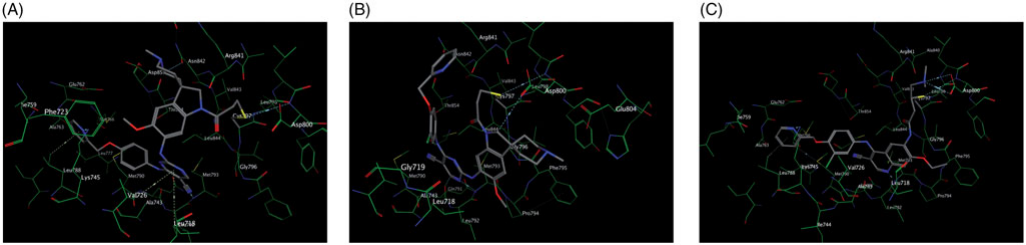
Docking of compounds the active site of 3W2Q: (A) Docking of A1 to the active site of 3W2Q. (B) Docking of A2 to the active site of 3W2Q (C) Docking of Neratinib to the active site of 3W2Q.

In this study, docking experiments were performed to elucidate the binding model of the compounds A1, A2 and neratinib with two mutant proteins 2jiv and 3w2q which was the crystal structure of EGFR kinase domain T790M mutation in compex with neratinib and EGFR kinase domain T790M/L858R mutant with neratinib, respectively. A1 and A2 could adopt the same combination mode as neratinib and bound to active sites of two proteins (shown in Figures 5 and 6). The binding modes of A1, A2 and neratinib with 2jiv showed that their binding energies (kcal/mol) is followed as Neratinib(–7.4)≈A1(–8.1)≈A2(–7.4), which is consistent with our further experimental activity with IC_50_ values of 0.17 μM, 0.089 μM and 0.13 μM. the 2-pyridinyl group of compounds were surrounded by hydrophobic residues in the expanded pocket, including Met-766 in the C-helix, Phe-856, and Met-790, the mutant gatekeeper residue, the nitrile substituent of neratinib also approaches the gatekeeper residue, and the covalent bond was formed between Cys-797 at the edge of the active site cleft and the crotonamide Michael-acceptor group on the inhibitor, it can not match the active site owing to the tension of the spiral ring, the piperidine ring on the spiral ring mainly pointed to the hydrophilic surface, which is far from the hinge region. These effecte could explain that the EGFR kinase adopted an inactive conformation (Figure 5). The binding modes of A1, A2 and neratinib with 3w2q showed that their binding energies (kcal/mol) is followed as Neratinib(–7.2)<A1(–8.3)≈A2(–7.7), which is consistent with our further experimental activity with IC_50_ values of 2.5 μM, 0.09 μM and 0.08 μM, A1 and A2 could bind in the ATP-binding cleft with a covalent to Cys-797 in a fashion similar to neratinib and the Cys-797 side-chain orientation wasrelevant to the position and length of the acceptor substituent to accommodate covalent binding, a distinct conformation change for Met790 was observed in the vicinity of these compounds, the side-chain rearrangement of Met790 was necessary to accommodate binding of the cyano group for A1, A2 and neratinib, The moiesty of 3-chloro-4-(pyridin-2-ylmethoxy)benzenamine in A1, A2 and neratinib had similar binding mode with Val726, Leu718, Leu788, and it can not match the active site owing to the tension of the spiral ring, the piperidine ring on the spiral ring mainly pointed to the hydrophilic surface, which is far from the hinge region (Figure 6).

## 3. Experimental part

### 3.1. General methods of synthesis

The solvents and reagents were purchased from commercial vendors and were dried and purified by conventional methods prior to use. Nuclear magnetic resonance (NMR) spectra were recorded on a Bruker AC-300P spectrometer with TMS as the internal standard and DMSO as solvent. ESI mass spectra were performed on an Shimadzu LCMS-2020. TLC analysis was carried out on silica gel 60 F254 silica plates (Merck, KGaA, Germany).

*Synthesis of benzyl5-methoxy-1,2-dihydrospiro[indole-3,4′-piperidine]-1′-carboxylate (* ***2*** *).* To a solution of (4-methoxyphenyl)hydrazine hydrochloride (9.85 g, 71.29 mmol) and benzyl 4-formylpiperidine-1-carboxylate (7 g, 28.31 mmol) in toluene/acetonitrile (100 ml/2 ml) was added trifluoroacetic acid (9.68 g, 85.63 mmol). The mixture was stirred for 12 h at 35 °C. The reaction was cooled to 0 °C and methanol (100 ml) was added followed by the slow addition of sodium borohydride (1.6 g, 42.29 mmol). After stirring for 3 h at 0–5 °C, the reaction was quenched by the addition of water (200 ml) and extracted with ethyl acetate (4 × 100 ml). The combined organic layer was dried over anhydrous sodium sulfate, filtered and concentrated under vacuum. The residue was purified by flash column chromatography with 0–30% ethyl acetate in petroleum ether to afford **2** (4.5 g, 45% yield). ^1^H NMR (300 MHz, DMSO-d_6_) δ 7.39–7.35 (m, 5H), 6.67 (d, *J* = 2.5 Hz, 1H), 6.57 – 6.49 (m, 1H), 6.42 (d, *J* = 8.4 Hz, 1H), 5.11 (m, 3H), 4.13 – 3.86 (m, 2H), 3.67 (s, 3H), 3.34 (s, 2H), 3.01–2.95 (m, 2H), 1.71–1.51 (m, 4H). MS (ESI) calculated for (C_21_H_24_N_2_O_3_) [M + 1]^+^, 353; found, 353.

*Synthesis of benzyl 5-methoxy-1-(trifluoroacetyl)-1,2-dihydrospiro[indole-3,4′- piperidine]-1′-carboxylate* ***(3)***. To a solution of **2** (4.3 g, 12.20 mmol) and triethylamine (2.44 g, 24.11 mmol) in dichloromethane (50 ml) was added trifluoroacetyl 2,2,2-trifluoroacetate (3.34 g, 15.90 mmol) slowly with stirring at 0 °C. The resulting solution was stirred for 12 h at room temperature under nitrogen. The reaction mixture was diluted with water (50 ml) and extracted with dichloromethane (3 × 40 ml). The combined organic layer was dried over anhydrous sodium sulfate, filtered and concentrated under vacuum. The residue was purified by flash column chromatography with 0–35% ethyl acetate in petroleum ether to afford **3** as a yellow solid (3.8 g, 66% yield). ^1^H NMR (300 MHz, DMSO-*d*_6_) δ 7.96 (d, *J* = 8.9 Hz, 1H), 7.44 – 7.27 (m, 5H), 7.05 (d, *J* = 2.6 Hz, 1H), 6.86 (dd, *J* = 8.9, 2.6 Hz, 1H), 5.11 (s, 2H), 4.20 (s, 2H), 4.06 – 3.99 (m, 2H), 3.76 (s, 3H), 3.01 (s, 2H), 1.85 (td, *J* = 13.2, 4.7 Hz, 2H), 1.65 (d, *J* = 13.0 Hz, 2H). MS (ESI) calculated for (C_23_H_23_F_3_N_2_O_4_) [M + 1]^+^, 449; found, 449.

*Synthesis of benzyl 5-methoxy-6-nitro-1-(trifluoroacetyl)-1,2-dihydrospiro[indole-3, 4′-piperidine]-1′-carboxylate (* ***4*** *).* To a solution of ***3***(4.1 g, 9.14 mmol) in acetic anhydride (50 ml) was added cupric nitrate trihydrate (2.43 g, 10 mmol) at 0 °C. After stirring for 3 h at room temperature, the reaction was quenched by the addition of water (50 ml) and extracted with dichloromethane (3 × 40 ml). The combined organic layer was dried over anhydrous sodium sulfate, filtered and concentrated under vacuum. The residue was purified by flash column chromatography with 0–80% ethyl acetate in petroleum ether to afford **4**as a yellow solid (3.9 g, 86% yield). ^1^H NMR (300 MHz, DMSO-*d*_6_) δ 8.47 (s, 1H), 7.57 (s, 1H), 7.52 – 7.23 (m, 5H), 5.12 (s, 2H), 4.29 (s, 2H), 4.16 – 4.03 (m, 2H), 4.00 (s, 3H), 3.05–2.96 (m, 2H), 1.99 – 1.91 (m, 2H), 1.73 (d, *J* = 13.2 Hz, 2H).

*Synthesis of 1-{6-amino-5-methoxy-1,2-dihydrospiro[indole-3,4′-piperidine]-1-yl}-2, 2,2-trifluoroethan-1-one (* ***5*** *).* To a solution of **4** (3.9 g, 7.90 mmol) in methanol (50 ml) was added palladium on activated carbon (500 mg, 10%). The mixture was stirred for 5 h at room temperature under hydrogen (2 atm). The solids were filtered out, and the filtrate was concentrated under vacuum to afford **5** (2.4 g, 92% yield). ^1^H NMR (300 MHz, DMSO-*d*_6_) δ 7.47 (s, 1H), 6.79 (s, 1H), 4.88 (s, 2H), 4.04 (s, 2H), 3.79 (s, 3H), 2.99 – 2.85 (m, 2H), 2.63 – 2.43 (m, 3H), 1.75 (td, *J* = 12.8, 4.3 Hz, 2H), 1.52 – 1.42 (m, 2H). MS (ESI) calculated for (C_15_H_18_F_3_N_3_O_2_) [M + 1]^+^, 330; found, 330.

*Synthesis of 1-{6-amino-5-methoxy-1′-methyl-1,2-dihydrospiro[indole-3,4′-piperidine]-1-yl}-2,2,2-trifluoroethan-1-one (* ***6*** *).* To a solution of **5** (2.4 g, 7.29 mmol) and triethylamine (2.2 g, 21.74 mmol) in tetrahydrofuran (40 ml) was added iodomethane (1.35 g, 9.5 mmol). After stirring for 6 h at room temperature, the mixture was concentrated under vacuum. The residue was purified by flash column chromatography with 0–10% methanol in dichloromethane to afford **6** as a light yellow solid (1.4 g, 58% yield). ^1^H NMR (300 MHz, DMSO-*d*_6_) δ 7.46 (s, 1H), 6.81 (s, 1H), 4.89 (s, 2H), 4.01 (s, 2H), 2.77 (d, *J* = 8.0 Hz, 2H), 2.21 (s, 3H), 1.96 – 1.79 (m, 4H), 1.53 (d, *J* = 10.5 Hz, 2H). MS (ESI) calculated for (C_16_H_20_F_3_N_3_O_2_) [M + 1]^+^, 344; found, 344.

*Synthesis of 2-{[5-methoxy-1′-methyl-1-(trifluoroacetyl)-1,2-dihydrospiro[indole-3, 4′-piperidine]-6-yl]amino}-4-{[4-(pyridin-2-ylmethoxy)phenyl]amino}pyrimidine-5-carbonitrile (* ***7a*** *).* To a solution of 2-chloro-4-[[4-(pyridin-2-ylmethoxy)phenyl]amino]pyrimidine -5-carbonitrile (300 mg, 0.89 mmol) and **6** (200 mg, 0.58 mmol) in 2-propanol (20 ml) was added *p*-toluenesulfonic acid (153 mg, 0.89 mmol) at room temperature. After was stirring for 2 h at 75 °C, the reaction mixture was diluted with saturated sodium bicarbonate aqueous solution (50 ml) and extracted with ethyl acetate (3 × 50 ml). The combined organic layer was dried over anhydrous sodium sulfate, filtered and concentrated under vacuum to afford **7a** as a yellow crude solid (370 mg, 72% purity, 71% yeild). MS (ESI) calculated for (C_33_H_31_F_3_N_8_O_3_) [M + 1]^+^, 645; found, 645.

Compound **7b-7d** was prepared in analogy to **7a**.

*Synthesis of 4-{[3-chloro-4-(pyridin-2-ylmethoxy)phenyl]amino}-2-{[5-methoxy-1*′*- methyl-1-(trifluoroacetyl)-1,2-dihydrospiro[indole-3,4*′*-piperidine]-6-yl]amino}pyrimidine-5-carbonitrile (* ***7b*** *) .* a yellow crude solid (290 mg, 65% purity, 63% yield). MS (ESI) calculated for (C_33_H_30_ClF_3_N_8_O_3_) [M + 1]^+^, 679; found, 679.

*Synthesis of 4-{[4-(benzyloxy)-3-chlorophenyl]amino}-2-{[5-methoxy-1*′*-methyl-1 -(trifluoroacetyl)-1,2-dihydrospiro[indole-3,4*′*-piperidine]-6-yl]amino}pyrimidine-5-carbonitrile (* ***7c*** *).* a yellow solid (210 mg, 70% purity, 53% yield). MS (ESI) calculated for (C_34_H_31_ClF_3_N_7_O_3_) [M + 1]^+^, 678; found, 678.

*Synthesis of 2-{[5-methoxy-1*′*-methyl-1-(trifluoroacetyl)-1,2-dihydrospiro[indole-3, 4*′*-piperidine]-6-yl]amino}-4-{[1-(pyridin-2-ylmethyl)-1H-pyrazol-4-yl]amino}pyrimidine-5-carbonitrile (* ***7d*** *).* a yellow solid (360 mg, 70% purity, 93% yield). MS (ESI) calculated for (C_30_H_29_F_3_N_10_O_2_) [M + 1]^+^, 619; found, 619.

*Synthesis of 2-({5-methoxy-1′-methyl-1,2-dihydrospiro[indole-3,4′-piperidine]-6-yl} amino)-4-{[4-(pyridin-2-ylmethoxy)phenyl]amino}pyrimidine-5-carbonitrile (* ***8a*** *).* To a solution of **7a** (370 mg, 0.40 mmol, 72% purity) in methanol (10 ml) was added potassium carbonate (280 mg, 2.03 mmol). After stirring for 2 h at room temperature, the mixture was diluted with water (50 ml) and extracted with ethyl acetate (30 ml × 3). The combined organic layer was dried over anhydrous sodium sulfate, filtered and concentrated under vacuum. The crude product was purified by Prep-HPLC [Column: XBridge Shield RP18 OBD Column, 5um, 19*150 mm; Mobile Phase A:Water with 10 mmol/L ammonium bicarbonate, Mobile Phase B: acetonitrile; Flow rate: 30 ml/min; Gradient: 10% B to 60% B in 13 min; 254 nm] to afford **8a** as a yellow solid (64 mg, 88.9% purity, 26% yield). ^1^H NMR (300 MHz, DMSO-*d*_6_) δ 9.25 (s, 1H), 8.58 (ddd, *J* = 4.9, 1.8, 1.0 Hz, 1H), 8.45 (s, 1H), 8.39 (s, 1H), 7.84 (td, *J* = 7.7, 1.8 Hz, 1H), 7.50–7.37 (m, 3H), 7.35 (ddd, *J* = 7.6, 4.8, 1.3 Hz, 1H), 7.00 – 6.91 (m, 2H), 6.83 (s, 1H), 6.79 (s, 1H), 5.15 (s, 2H), 4.91 (s, 1H), 3.68 (s, 3H), 3.24 (s, 2H), 2.78 – 2.60 (m, 2H), 2.20 (s, 3H), 1.98 (t, *J* = 11.7 Hz, 2H), 1.90 – 1.73 (m, 2H), 1.61 – 1.44 (m, 2H).MS (ESI) calculated for (C_31_H_32_N_8_O_2_) [M + 1]^+^, 549; found, 549.

Compound **8b-8d** was prepared in analogy to **8a**.

*Synthesis of 4-{[3-chloro-4-(pyridin-2-ylmethoxy)phenyl]amino}-2-({5-methoxy-1*′ *-methyl-1,2-dihydrospiro[indole-3,4*′*-piperidine]-6-yl}amino)pyrimidine-5-carbonitrile (* ***8b*** *).* a yellow solid (46 mg, 85.6% purity, 29% yield). ^1^H NMR (300 MHz, DMSO-*d*_6_) δ 9.31 (s, 1H), 8.69 – 8.54 (m, 2H), 8.41 (s, 1H), 7.87 (td, *J* = 7.7, 1.8 Hz, 1H), 7.64 (s, 1H), 7.58 – 7.54 (m, 2H), 7.42 – 7.30 (m, 1H), 7.14 (d, *J* = 9.0 Hz, 1H), 6.81 (s, 1H), 6.74 (s, 1H), 5.25 (s, 2H), 4.94 (s, 1H), 3.68 (s, 3H), 3.24 (s, 2H), 2.67 (d, *J* = 10.5 Hz, 2H), 2.17 (s, 3H), 2.00 – 1.71 (m, 4H), 1.52 (d, *J* = 11.8 Hz, 2H).MS (ESI) calculated for (C_31_H_31_ClN_8_O_2_) [M + 1]^+^, 583; found, 583.

*Synthesis of 4-{[4-(benzyloxy)-3-chlorophenyl]amino}-2-({5-methoxy-1*′*-methyl-1,2 -dihydrospiro[indole-3,4*′*-piperidine]-6-yl}amino)pyrimidine-5-carbonitrile (* ***8c*** *).* a yellow solid (70 mg, 60% yield). ^1^H NMR (300 MHz, DMSO-*d*_6_) δ 9.30 (s, 1H), 8.60 (s, 1H), 8.41 (s, 1H), 7.63 (s, 1H), 7.59 – 7.51 (m, 1H), 7.50 – 7.30 (m, 5H), 7.15 (d, *J* = 9.0 Hz, 1H), 6.81 (s, 1H), 6.75 (s, 1H), 5.17 (s, 2H), 4.94 (s, 1H), 3.68 (s, 3H), 3.28 – 3.19 (m, 2H), 2.68 (d, *J* = 10.7 Hz, 2H), 2.17 (s, 3H), 2.02 – 1.75 (m, 4H), 1.53 (d, *J* = 12.0 Hz, 2H).MS (ESI) calculated for (C_32_H_32_ClN_7_O_2_) [M + 1]^+^, 582; found, 582.

*Synthesis of 2-({5-methoxy-1*′*-methyl-1,2-dihydrospiro[indole-3,4*′*-piperidine]-6-yl} amino)-4-{[1-(pyridin-2-ylmethyl)-1H-pyrazol-4-yl]amino}pyrimidine-5-carbonitrile (* ***8d*** *).* a yellow solid (67 mg, 34% yield). ^1^H NMR (300 MHz, DMSO-*d*_6_) δ 9.64 (s, 1H), 8.81 (s, 1H), 8.51 (dd, *J* = 5.0, 1.6 Hz, 1H), 8.36 (s, 1H), 7.80 – 7.55 (m, 2H), 7.34 – 7.22 (m, 1H), 6.86 (s, 1H), 6.79 (s, 1H), 6.74 (s, 1H), 5.31 (s, 2H), 5.15 (s, 1H), 3.65 (s, 3H), 3.20 (s, 2H), 2.63 (d, *J* = 10.9 Hz, 2H), 2.17 (s, 3H), 1.90 (t, *J* = 11.6 Hz, 2H), 1.75 (t, *J* = 12.5 Hz, 2H), 1.43 (d, *J* = 12.4 Hz, 2H). MS (ESI) calculated for (C_28_H_30_N_10_O) [M + 1]^+^, 523; found, 523.

*Synthesis of 5′-methoxy-1′, 2′-dihydrospiro [cyclopropane-1, 3′-indole]-2′-one (* ***10*** *).* To a solution of *5-methoxy-2, 3-dihydro-1H-indol-2-one* (**9**, 5 g, 30.64 mmol,) and diisopropylamine (6.14 g, 60.79 mmol) in dry tetrahydrofuran (50 ml) was added n-butyllithium (49 ml, 122.68 mmol, 2.5 M in hexane) slowly at -60 °C. The mixture was stirred for 1 h at –60 °C. This was followed by the addition of 1, 2-dibromoethane (6.89 g, 36.68 mmol) slowly at –60 °C. The resulting solution was warmed to room temperature for 14 h. The reaction was then quenched by the addition of 100 ml of water and extracted with ethyl acetate (3 × 300 ml). The combined organic layer was dried over anhydrous sodium sulfate, filtered and concentrated under vacuum. The residue was purified by flash column chromatography with 0 ∼ 50% ethyl acetate in petroleum ether to afford **10** as an off-white solid (2.5 g, 43% yield). ^1^H NMR (300 MHz, CDCl_3_) δ 8.89 (s, 1H), 6.89 (d, *J* = 9.6 Hz, 1H), 6.86 (dd, *J_1_* = 8.7 Hz, *J_2_* = 2.7 Hz, 1H), 6.47 (d, *J* = 2.4 Hz, 1H), 3.77 (s, 3H), 1.78–1.74 (m, 2H), 1.53–1.49 (m, 2H). MS (ESI) calc’d for (C_11_H_11_NO_2_) [M + 1]^+^, 190; found, 190.

*Synthesis of 5′-methoxy-6′-nitro-1′,2′-dihydrospiro[cyclopropane-1,3′-indole]-2′-one (* ***11*** *).* To a solution of **10** (2.5 g, 13.21 mmol) and acetyl acetate (2.0 g, 19.59 mmol) in dichloromethane (30 ml) was added nitric acid (1.5 g, 23.80 mmol). The resulting solution was stirred for 2 h at room temperature. The reaction was quenched by the addition of saturated sodium bicarbonate aqueous solution (20 ml). The solids were collected by filtration and dried in an oven under reduced pressure to afford **11** as a yellow solid (1.7 g, 55% yield). H^1^ NMR (300 MHz, DMSO-*d_6_*) δ 10.68 (s, 1H), 7.37 (s, 1H), 7.13 (s, 1H), 3.87 (s, 3H), 1.79–1.71 (m, 2H), 1.61–1.58 (m, 2H).MS (ESI) calc’d for (C_11_H_10_N_2_O_4_) [M + 1] ^+^, 235; found, 235.

*Synthesis of 6′-amino-5′-methoxy-1′,2′-dihydrospiro[cyclopropane-1,3′-indole]-2′-one (* ***12*** *).* A degassed solution of **11** (1.7 g, 7.26 mmol), iron dust (1.6 g, 28.57 mmol) and ammonium chloride (1.9 g, 35.85 mmol) in methanol and water (1:1) (10 ml) was stirred for 6 h at 70 °C. The reaction mixture was diluted with water (20 ml) and extracted with ethyl acetate (3 × 50 ml). The combined organic layer was dried over anhydrous sodium sulfate, filtered and concentrated under vacuum to afford **12** as a yellow solid (1.1 g, 74% yield). H^1^ NMR (400 MHz, DMSO-*d_6_*) δ 10.07 (s, 1H), 6.50 (s, 1H), 6.34 (s, 1H), 4.68 (s, 2H), 3.69 (s, 3H), 1.38–1.35 (m, 2H), 1.32–1.30 (m, 2H). MS (ESI) calc’d for (C_11_H_12_N_2_O_2_) [M + 1]^+^, 205; found, 205.

*Synthesis of 5′-methoxy-1′,2′-dihydrospiro[cyclopropane-1,3′-indole]-6′-amine (* ***13*** *).* To a solution of **12** (500 mg, 2.45 mmol) in dry tetrahydrofuran (10 ml) was added lithium aluminum hydride (375 mg, 9.87 mmol) at room temperature. The resulting solution was stirred for 4 h at 70 °C. The reaction was then quenched by the addition of water (20 ml) and extracted with ethyl acetate (3 × 50 ml). The combined organic layer was dried over anhydrous sodium sulfate filtered and concentrated under vacuum. The residue was purified by flash column chromatography with 0 ∼ 50% ethyl acetate in petroleum ether to afford **13** as a brown solid (260 mg, 56% yield). ^1^H NMR (300 MHz, DMSO-*d_6_*) δ 6.14 (s, 1H), 5.94 (s, 1H), 4.92 (s, 1H), 4.04 (s, 2H), 3.61 (s, 3H), 3.31 (s, 2H), 0.80–0.72 (m, 4H). MS (ESI) calc’d for (C_11_H_14_N_2_O) [M + 1]^+^, 191; found, 191.

*Synthesis of 5-bromo-2-N-{5′-methoxy-1′, 2′-dihydrospiro [cyclopropane-1, 3′-indole]-6′-yl}-4-N-[4-(pyridin-2-ylmethoxy) phenyl] pyrimidine-2,4-diamine (* ***14a*** *).* To a solution of **13** (450 mg, 2.37 mmol) and 5-bromo-2-chloro-N-[4-(pyridin-2-ylmethoxy)phenyl]pyrimidin-4-amine (647 mg, 1.65 mmol) in dioxone (10 ml) was added 4-methylbenzene-1-sulfonic acid monohydrate (408 mg, 2.37 mmol). The resulting solution was stirred for 6 h at 100 °C. The reaction mixture was diluted water (20 ml) and extracted with ethyl acetate (3 × 30 ml). The combined organic layer was dried over sodium sulfate, filtered and concentrated under vacuum. The residue was purified by flash column chromatography with 0 ∼ 50% ethyl acetate in petroleum ether to afford **14a** as an off-white solid (260 mg, 20% yield). ^1^H NMR (300 MHz, DMSO-*d_6_*) δ 8.59 (d, *J* = 4.5 Hz, 1H), 8.51 (s, 1H), 8.17 (s, 1H), 7.87 (dd, *J*_1_= 7.5 Hz, 1.8 Hz, 1H), 7.56–7.47 (m, 3H), 7.37–7.33 (m, 2H), 7.07 (d, *J* = 8.7 Hz, 1H), 6.26 (s, 1H), 5.18 (s, 2H), 4.18–4.01 (br, 1H), 3.93 (s, 2H), 3.67 (s, 3H), 0.94 (d, *J* = 3.0 Hz, 4H). MS (ESI) calc’d for (C_27_H_25_BrN_6_O_2_) [M + 1]^+^, 545, 547; found, 545, 547.

Compound **14b-14d** was prepared in analogy to 14**a**.

*Synthesis of 5-bromo-4-N-[3-chloro-4-(pyridin-2-ylmethoxy)phenyl]-2-N-{5*′*-methoxy -1*′*,2*′*-dihydrospiro[cyclopropane-1,3*′*-indole]-6*′*-yl}pyrimidine-2,4-diamine (* **14 b** *).* an off-white solid (11.5 mg, 8% yield). ^1^H NMR (400 MHz, DMSO-*d_6_*) δ 8.65–8.60 (m, 2H), 8.23 (s, 1H), 7.91–7.87 (m, 2H), 7.61–7.54 (m, 2H), 7.39–7.36 (m, 1H), 7.26–7.24 (m, 1H), 6.30 (s, 1H), 5.28 (s, 2H), 4.20 (br, 1H), 3.99 (s, 2H), 3.69 (s, 3H), 0.95 (d, *J* = 3.0 Hz, 4H). MS (ESI) calc’d for (C_27_H_24_BrClN_6_O_2_) [M + 1]^+^, 579, 581; found, 579, 581.

*Synthesis of 4-N-[4-(benzyloxy)-3-chlorophenyl]-5-bromo-2-N-{5*′*-methoxy-1*′*,2*′*- dihydrospiro[cyclopropane-1,3*′*-indole]-6*′*-yl}pyrimidine-2,4-diamine (* ***14c*** *).* An off-white solid (14.6 mg, 10% yield). ^1^H NMR (400 MHz, DMSO-*d_6_*) δ 8.63 (s, 1H), 8.22 (s, 1H), 7.85 (s, 1H), 7.54 (d, J = 8.3 Hz, 1H), 7.49 (d, J = 7.5 Hz, 2H), 7.42 (t, J = 7.5 Hz, 2H), 7.35 (t, J = 7.2 Hz, 1H), 7.25 (d, J = 8.9 Hz, 1H), 6.29 (s, 1H), 5.20 (s, 2H), 4.32 (s, 3H), 3.97 (s, 2H), 3.68 (s, 3H), 0.95 (d, J = 9.8 Hz, 4H).MS (ESI) calc’d for (C_28_H_25_BrClN_5_O_2_) [M + 1]^+^, 578, 580; found, 578, 580.

*Synthesis of 5-bromo-2-N-{5′-methoxy-1′, 2′-dihydrospiro [cyclopropane-1, 3′-indole] -6′-yl}-4-N-[1-(pyridin-2-ylmethyl)-1H-pyrazol-4-yl] pyrimidine-2, 4-diamine (* ***14d*** *).* An off-white solid (260 mg, 32% yield) of. ^1^H NMR (300 MHz, DMSO-*d_6_*) δ 8.80 (s, 1H), 8.54 (d, *J* = 4.8 Hz, 1H), 8.14 (s, 1H), 8.09 (s, 1H), 7.79–7.73 (m, 2H), 7.62 (s, 1H), 7.34–7.29 (m, 1H), 7.07 (d, *J* = 7.8 Hz, 1H), 6.28 (s, 1H), 5.41 (s, 2H), 4.46 (br, 2H), 4.00 (s, 2H), 3.68 (s, 3H), 0.94–0.93 (m, 4H). MS (ESI) calc’d for (C_24_H_23_BrN_8_O) [M + 1]^+^, 519, 521; found, 519, 521.

*Synthesis of 2-({5′-methoxy-1′, 2′-dihydrospiro [cyclopropane-1, 3′-indole]-6′-yl} amino)-4-{[4-(pyridin-2-ylmethoxy) phenyl] amino} pyrimidine-5-carbonitrile (* ***15a*** *).* A degassed solution of **14a** (200 mg, 0.37 mmol), zincdicarbonitrile (214 mg, 1.82 mmol), tris(dibenzylideneacetone)dipalladium (39 mg, 0.04 mmol) and 1,1′-ferrocenebis(diphenylphosphine) (41 mg, 0.07 mmol) in *N,N*-dimethylformamide and water (10:1) (2 ml) was stirred for 3 h at 110 °C in microwave reactor. The reaction mixture was diluted with water (25 ml) and extracted with ethyl acetate (3 × 15 ml). The combined organic layer was dried over sodium sulfate, filtered and concentrated under vacuum. The residue was purified by flash column chromatography with 0 ∼ 50% ethyl acetate in petroleum ether to afford **15a** as a brown solid (110 mg, 55% yield). ^1^H NMR (300 MHz, DMSO-*d_6_*) δ 9.35 (br, 1H), 8.59 (d, *J* = 4.5 Hz, 1H), 8.46 (s, 1H), 7.87 (dd, *J*_1_= 7.8, 1.5 Hz, 1H), 7.55(br, 2H), 7.37–7.33 (m, 2H), 7.16–7.01 (m, 3H), 6.29 (s, 1H), 5.18 (s, 2H), 4.65 (s, 1H), 4.04–3.97 (m, 2H), 3.75 (br, 1H), 3.69 (s, 3H), 1.00–0.95 (m, 4H). MS (ESI) calc’d for (C_28_H_25_N_7_O_2_) [M + 1]^+^, 492; found, 492.

Compound **15b-15d** was prepared in analogy to 15**a**

*Synthesis of 4-[[3-chloro-4-(pyridin-2-ylmethoxy)phenyl]amino]-2-([5′-methoxy-1′,2′ -dihydrospiro[cyclopropane-1,3′-indole]-6′-yl]amino)pyrimidine-5-carbonitrile (* ***15b*** *).*

a yellow solid (5.3 mg, 12% yield). ^1^H NMR (300 MHz, DMSO-*d_6_*) δ 8.60–8.50 (m, 2H), 7.96–7.36 (m, 6H), 7.24–7.10 (m, 1H), 6.33 (s, 1H), 5.27 (s, 2H), 4.17–4.07 (m, 2H), 3.71 (br, 3H), 3.69 (s, 3H), 1.04–0.98 (m, 4H). MS (ESI) calc’d for (C_28_H_24_ClN_7_O_2_) [M + 1]^+^, 526; found, 526.

*Synthesis of 4-{[4-(benzyloxy)-3-chlorophenyl]amino}-2-({5′-methoxy-1′,2′- dihydrospiro[cyclopropane-1,3′-indole]-6′-yl}amino)pyrimidine-5-carbonitrile (* ***15c*** *).* A yellow solid(4.2 mg, 9% yield). ^1^H NMR (300 MHz, DMSO-*d_6_*) δ 9.15 (s, 1H), 8.46 (s, 1H), 7.75 (s, 1H), 7.49–7.22 (s, 8H), 6.30 (s, 1H), 5.20 (s, 2H), 4.17–4.07 (m, 2H), 4.04 (s, 2H), 3.71 (s, 3H), 0.97 (s, 4H). MS (ESI) calc’d for (C_29_H_25_ClN_6_O_2_) [M + 1]^+^, 525; found, 525.

*Synthesis of 2-({5*′*-methoxy-1*′*, 2*′*-dihydrospiro [cyclopropane-1, 3*′*-indole]-6*′*-yl} amino)-4-{[1-(pyridin-2-ylmethyl)-1H-pyrazol-4-yl] amino} pyrimidine-5-carbonitrile (* ***15d*** *).* a yellow solid (110 mg, 68% yield). ^1^H NMR (300 MHz, DMSO-*d_6_*) δ 9.67 (s, 1H), 8.54 (d, *J* = 3.6 Hz, 1H), 8.46 (s, 1H), 8.09 (s, 1H), 7.79–7.74 (m, 3H), 7.34–7.30 (m, 1H), 7.07–7.05 (m, 1H), 6.32 (s, 1H), 5.42 (s, 2H), 4.66 (br, 2H), 4.08 (s, 2H), 3.71 (s, 3H), 0.97 (s, 4H). MS (ESI) calc’d for (C_25_H_23_N_9_O) [M + 1]^+^, 466; found, 466.

*Synthesis of methyl 1–(5-methoxy-2,4-dinitrophenoxy)cyclopropanecarboxylate (* ***17*** *).* To a solution of *1-fluoro-5-methoxy-2,4-dinitrobenzene* (**16**, 20 g, 92.54 mmol) and *methyl 1-hydroxycyclopropane-1-carboxylate* (11.8 g, 101.62 mmol) in acetonitrile (400 ml) was added cesium carbonate (45.14 g, 138.9 mmol) at room temperature. The resulting solution was stirred for 12 h at room temperature. The reaction mixture was diluted with water (400 ml) and extracted with ethyl acetate (200 ml × 3). The combined organic phase was washed with brine (200 ml × 3), then dried over anhydrous magnesium sulfate, filtered and concentrated under reduced pressure. The residue was purified by flash column chromatography with 0 ∼ 50% ethyl acetate in petroleum ether to afford **17** as yellow solid (11 g, 38% yield). ^1^H NMR (300 MHz, DMSO-*d_6_*) δ 8.70 (s, 1H), 6.88 (s, 1H), 4.11 (s, 3H), 3.71 (s, 3H), 1.73 (t, *J* = 5.8 Hz, 2H), 1.53 (t, *J* = 5.8 Hz, 2H). MS (ESI) calc’d for (C_12_H_12_N_2_O_8_) [M + 1]^+^, 313; found, 313.

*Synthesis of 6-amino-7-methoxy-3,4-dihydrospiro[1,4-benzoxazine-2,1′- cyclopropane]-3-one (* ***18*** *).* A mixture of **17** (10 g, 32.03 mmol), iron dust (5.4 g, 96.70 mmol) and ammonium chloride (10.1 g, 188.8 mmol) in methanol (150 ml) and water (150 ml) was stirred for 12 h at 70 °C. The resulting solution was extracted with ethyl acetate (300 ml × 2). The combined organic phase was washed with brine (200 ml × 3), then dried over anhydrous magnesium sulfate, filtered and the filtrate was concentrated under reduced pressure. The residue was purified by flash column chromatography with 20 ∼ 50% ethyl acetate in petroleum ether to afford **18** as a yellow solid (4 g, 57% yield). ^1^H NMR (400 MHz, DMSO-*d_6_*) δ 10.38 (s, 1H), 6.44 (s, 1H), 6.28 (s, 1H), 4.53 (s, 2H), 3.68 (s, 3H), 1.16 (t, *J* = 3.0 Hz, 2H), 1.06 (t, *J* = 3.0 Hz, 2H). MS (ESI) calc’d for (C_11_H_12_N_2_O_3_) [M + 1]^+^, 221; found, 221.

*Synthesis of 7-methoxy-3,4-dihydrospiro[1,4-benzoxazine-2,1′-cyclopropane]-6-amine (* ***19*** *).* To a solution of **18** (1 g, 4.54 mmol) in dry tetrahydrofuran (20 ml) was added lithium aluminum hydride (690 mg, 18.18 mmol,) at 0 °C. Then the resulting solution was heated to 70 °C for 2 h. The reaction was then quenched by the addition of 50 ml of water at 0 °C and extracted with ethyl acetate (100 ml × 2). The combined organic phase was washed with brine (100 ml × 2), then dried over anhydrous magnesium sulfate, filtered and the filtrate was concentrated under reduced pressure. The residue was purified by flash column chromatography with ethyl acetate to afford **19** as brown oil (0.5 g, 53% yield). ^1^H NMR (300 MHz, DMSO-*d_6_*) δ 6.17 (s, 1H), 6.00 (s, 1H), 5.14 (s, 1H), 4.11 (s, 2H), 3.59 (s, 3H), 3.06 (d, *J* = 2.1 Hz, 2H), 0.79–0.77 (m, 2H), 0.63–0.59 (m, 2H). MS (ESI) calc’d for (C_11_H_14_N_2_O_2_) [M + 1]^+^, 207; found, 207.

*Synthesis of 5-bromo-2-N-{7-methoxy-3,4-dihydrospiro[1,4-benzoxazine-2,1′- cyclopropane]-6-yl}-4-N-[4-(pyridin-2-ylmethoxy)phenyl]pyrimidine-2,4-diamine (* ***20a*** *).* To a solution of 5-bromo-2-chloro-N-[4-(pyridin-2-ylmethoxy)phenyl]pyrimidin -4-amine (151.5 mg, 0.39 mmol) and **19** (100 mg, 0.48 mmol) in dioxane (5 ml) was added 4-Toluene sulfonic acid monohydrate (83.5 mg, 0.49 mmol) at room temperature. The resulting solution was stirred for 2 h at 90 °C. The reaction mixture was diluted with saturated sodium bicarbonate solution (30 ml) and extracted with ethyl acetate (50 ml × 3). The combined organic phase was washed with brine (50 ml × 3), then dried over anhydrous magnesium sulfate, filtered and the filtrate was concentrated under reduced pressure. The residue was purified by flash column chromatography with 0–10% ethyl acetate in methanol to afford **20a** as an off-white solid (59.2 mg, 22% yield). ^1^H NMR (300 MHz, DMSO-*d_6_*) δ 8.59 (d, *J* = 4.5 Hz, 1H), 8.35 (s, 1H), 8.07 (s, 1H), 7.87 (dd, *J*_1_= 7.5 Hz, *J_2_* = 1.8 Hz, 1H), 7.70 (s, 1H), 7.55–7.45 (m, 3H), 7.39–7.31 (m, 1H), 6.99–6.93 (m, 3H), 6.36 (s, 1H), 5.16–5.12 (m, 3H), 3.62 (s, 3H), 3.13 (d, J = 2.8 Hz, 2H), 0.88–0.84 (m, 2H), 0.69–0.65 (m, 2H) MS (ESI) calc’d for (C_27_H_25_BrN_6_O_3_) [M + 1]^+^, 561, 563; found, 561, 563.

Compound **20b-20d** was prepared in analogy to 20**a**

*Synthesis of 5-bromo-4-N-[3-chloro-4-(pyridin-2-ylmethoxy)phenyl]-2-N-{7-methoxy -3,4-dihydrospiro[1,4-benzoxazine-2,1*′*-cyclopropane]-6-yl}pyrimidine-2,4-diamine* (**20 b**). a gray solid (57.7 mg, 20% yield). ^1^H NMR (300 MHz, DMSO-*d_6_*) δ 8.59 (d, *J* = 2.8 Hz, 1H), 8.44 (s, 1H), 8.10 (s, 1H), 7.91–7.84 (m, 2H), 7.71 (s, 1H), 7.64–7.55 (m, 2H), 7.39–7.35 (m, 1H), 7.17 (d, *J* = 6.0 Hz, 1H), 6.92 (s, 1H), 6.37 (s, 1H), 5.27–5.15 (m, 3H), 3.61 (s, 3H), 3.12 (d, J = 2.8 Hz, 2H), 0.88–0.84 (m, 2H), 0.68–0.64 (m, 2H). MS (ESI) calc’d for (C_27_H_24_BrClN_6_O_3_) [M + 1]^+^, 595, 597; found, 595, 597.

*Synthesis of 4-N-[4-(benzyloxy)-3-chlorophenyl]-5-bromo-2-N-{7-methoxy-3,4- dihydrospiro[1,4-benzoxazine-2,1′-cyclopropane]-6-yl}pyrimidine-2,4-diamine (* ***20c*** *).* An off-white solid(28.9 mg, 20% yield). ^1^H NMR (300 MHz, DMSO-*d_6_*) δ 8.41 (s, 1H), 8.10 (s, 1H), 7.83 (s, 1H), 7.71 (s, 1H), 7.62–7.60 (m, 1H), 7.48–7.33 (m, 5H), 7.15 (d, *J* = 6.0 Hz, 1H), 6.93 (s, 1H), 6.38 (s, 1H), 5.20–5.18 (m, 3H), 3.62 (s, 3H), 3.14 (d, J = 2.8 Hz, 2H), 0.88–0.84 (m, 2H), 0.68–0.64 (m, 2H). MS (ESI) calc’d for (C_28_H_25_BrClN_5_O_3_) [M + 1]^+^, 594, 596; found, 594, 596.

*Synthesis of 5-bromo-2-N-{7-methoxy-3,4-dihydrospiro[1,4-benzoxazine-2,1*′ *-cyclopropane]-6-yl}-4-N-[1-(pyridin-2-ylmethyl)-1H-pyrazol-4-yl]pyrimidine-2,4-diamine* (**20d**). A gray solid (42.9 mg, 24% yield). ^1^H NMR (300 MHz, DMSO-*d_6_*) δ 8.78 (s, 1H), 8.52–8.50 (m, 1H), 8.16–8.12 (m, 1H), 7.98 (s, 1H), 7.93 (s, 1H), 7.76–7.69 (s, 2H), 7.31–7.27 (m, 1H), 6.89–6.87 (m, 2H), 6.34 (s, 1H), 5.37–5.31 (m, 3H), 3.64 (s, 3H), 3.11 (d, J = 2.8 Hz, 2H), 0.88–0.84 (m, 2H), 0.69–0.65 (m, 2H). MS (ESI) calc’d for (C_24_H_23_BrN_8_O_2_) [M + 1]^+^, 535, 537; found, 535, 567.

*Synthesis of 2-({7-methoxy-3,4-dihydrospiro[1,4-benzoxazine-2,1′-cyclopropane] -6-yl}amino)-4-{[4-(pyridin-2-ylmethoxy)phenyl]amino}pyrimidine-5-carbonitrile (* ***21a*** *).* A degassed solution of **20a** (30 mg, 0.05 mmol) tris(dibenzylideneacetone)dipalladium (11.1 mg, 0.01 mmol), zinc cyanide (22.7 mg, 0.19 mmol) and 1,1′-bis(diphenylphosphino)ferrocene (11.9 mg, 0.02 mmol) in *N,N*-dimethylformamide (1 ml) and water (0.1 ml) was stirred for 3 h at 110 °C in microwave reactor. The resulting solution was diluted with water (20 ml) and extracted with ethyl acetate (30 ml × 2). The combined organic phase was washed with brine (50 ml × 2), then dried over anhydrous magnesium sulfate, filtered and the filtrate was concentrated under reduced pressure. The residue was purified by flash column chromatography with 0–10% methanol in ethyl acetate to afford **21a** as a yellow solid(13.9 mg, 51% yield). ^1^H NMR (400 MHz, DMSO-*d_6_*) δ 9.16 (br, 1H), 8.70–8.60 (m, 2H), 8.36 (s, 1H), 7.87 (dd, *J*_1_= 7.8 Hz, *J_2_* = 1.5 Hz, 1H), 7.55–7.43(m, 3H), 7.39–7.31 (m, 1H), 6.97–6.93 (m, 2H), 6.80–6.71 (m, 1H), 6.38 (s, 1H), 5.23–5.14 (m, 3H), 3.59 (s, 3H), 3.15 (s, 2H), 0.89–0.87 (m, 2H), 0.70–0.67 (m, 2H). MS (ESI) calc’d for (C_28_H_25_N_7_O_3_) [M + 1]^+^, 508; found, 508.

Compound **21 b-21d** was prepared in analogy to **21a**

*Synthesis of 4-[[3-chloro-4-(pyridin-2-ylmethoxy)phenyl]amino]-2-([7-methoxy-3,4 -dihydrospiro[1,4-benzoxazine-2,1*′*-yclopropane]-6-yl]amino)pyrimidine-5-carbonitrile (* ***21 b*** *).* A yellow solid (20.7 mg, 23% yield). ^1^H NMR (300 MHz, DMSO-*d_6_*) δ 9.23 (br, 1H), 8.74 (br, 1H), 8.58 (d, *J* = 2.1 Hz, 1H), 8.39 (s, 1H), 7.90–7.85 (m, 1H), 7.72–7.50 (m, 3H), 7.38–7.32 (m, 1H), 7.13–7.09 (m, 1H), 6.79–6.71 (m, 1H), 6.40 (s, 1H), 5.40–5.24 (m, 3H), 3.60 (s, 3H), 3.13 (s, 2H), 0.89–0.87 (m, 2H), 0.69–0.67 (m, 2H). MS (ESI) calc’d for (C_28_H_24_ClN_7_O_3_) [M + 1]^+^, 542; found, 542.

*Synthesis of 4-[[4-(benzyloxy)-3-chlorophenyl]amino]-2-([7-methoxy-3,4- dihydrospiro[1,4-benzoxazine-2,1*′*-cyclopropane]-6-yl]amino)pyrimidine-5-carbonitrile (* ***21c*** *).* A yellow solid (21.6 mg, 24% yield). ^1^H NMR (400 MHz, DMSO-*d_6_*) δ 8.82 (br, 2H), 8.39 (s, 1H), 7.69 (br, 1H), 7.55 (s, 1H), 7.48–7.36 (m, 5H), 7.13–7.09 (m, 1H), 6.72–6.69 (m, 1H), 6.41 (s, 1H), 5.30–5.16 (m, 3H), 3.60 (s, 3H), 3.15 (s, 2H), 0.89–0.87 (m, 2H), 0.69–0.67 (m, 2H). MS (ESI) calc’d for (C_29_H_25_ClN_6_O_3_) [M + 1]^+^, 541; found, 541.

*Synthesis of 2-([7-methoxy-3,4-dihydrospiro[1,4-benzoxazine-2,1*′*-cyclopropane]-6- yl]amino)-4-[[1-(pyridin-2-ylmethyl)-1H-pyrazol-4-yl]amino]pyrimidine-5-carbonitrile (* ***21d*** *).* A yellow solid (21.8 mg, 48% yield). ^1^H NMR (400 MHz, DMSO-*d_6_*) δ 9.62 (br, 1H), 8.84 (s, 1H), 8.51 (d, *J* = 2.1 Hz, 1H), 8.33 (s, 1H), 7.80–7.70(m, 2H), 7.65–7.50 (m, 1H), 7.32–7.29 (m, 1H), 6.90–6.82 (m, 1H), 6.80–6.73 (m, 1H), 6.37 (s, 1H), 5.54 (s, 1H), 5.28 (br, 2H), 3.56 (s, 3H), 3.07 (s, 2H), 0.89–0.87 (m, 2H), 0.70–0.67 (m, 2H). MS (ESI) calc’d for (C_25_H_23_N_9_O_2_) [M + 1]^+^, 482; found, 482.

*Synthesis of 2-{[5-methoxy-1′-methyl-1-(prop-2-enoyl)-1,2-dihydrospiro[indole-3,4′ -piperidine]-6-yl]amino}-4-{[4-(pyridin-2-ylmethoxy)phenyl]amino}pyrimidine-5-carbonitrile (* ***A1*** *).* To a solution of **8a** (42 mg, 0.08 mmol) and triethylamine (80 mg, 0.79 mmol) in dichloromethane (5 ml). This was followed by the addition of a solution of prop-2-enoyl chloride (5.6 mg, 0.06 mmol) in dichloromethane (0.2 ml) at –20 °C under nitrogen. After stirring for 1 h at –20 °C, the mixture was concentrated under vacuum. The crude product was purified by Prep-HPLC [Column: XBridge Shield RP18 OBD Column, 5um, 19*150 mm;: Water with 10 mmol/L ammonium bicarbonate, mobile phase B: acetonitrile. Gradient: 25.0%B up to 48.0%B in 10 min; Detector: UV 220 nm] to afford **A1** as a white solid (14.4 mg, 31% yield). ^1^H NMR (300 MHz, DMSO-*d*_6_) δ 10.17 (s, 1H), 9.19 (s, 1H), 9.00 (s, 1H), 8.60 (d, *J* = 4.2 Hz, 1H), 8.40 (s, 1H), 8.24 (s, 1H), 7.84 (td, *J* = 7.8, 1.8 Hz, 1H), 7.50–7.34 (m, 4H), 6.81 – 6.71 (m, 3H), 6.30 (d, *J* = 18.0 Hz, 1H), 5.84 (d, *J* = 11.1 Hz, 1H), 5.09 (s, 2H), 4.23 (s, 2H), 3.74 (s, 3H), 3.47 – 3.43 (s, 2H), 3.20 – 3.13 (m, 2H), 2.80 (s, 3H), 2.24 (t, *J* = 12.0 Hz, 2H), 1.89 – 1.85 (m, 2H).MS (ESI) calculated for (C_34_H_34_N_8_O_3_) [M + 1]^+^, 603; found, 603.

Compound **A2-A4** was prepared in analogy to **A1**.

*Synthesis of 4-{[3-chloro-4-(pyridin-2-ylmethoxy)phenyl]amino}-2-{[5-methoxy-1*′*- methyl-1-(prop-2-enoyl)-1,2-dihydrospiro[indole-3,4*′*-piperidine]-6-yl]amino}pyrimidine-5-carbonitrile (* ***A2*** *).* A white solid (4.2 mg, 12% yield). ^1^H NMR (300 MHz, Methanol-*d*_4_) δ 8.64 (s, 1H), 8.57 (dt, *J* = 4.8, 1.4 Hz, 1H), 8.35 (s, 1H), 7.91 (dd, *J* = 8.5, 6.7 Hz, 1H), 7.73 – 7.64 (m, 2H), 7.46 – 7.29 (m, 2H), 6.97– 6.94 (m, 2H), 6.77 (dd, *J* = 16.7, 10.3 Hz, 1H), 6.30 (d, *J* = 16.8 Hz, 1H), 5.77 (d, *J* = 10.6 Hz, 1H), 5.16 (s, 2H), 4.12 (s, 2H), 3.90 (s, 3H), 2.93 (d, *J* = 12.0 Hz, 2H), 2.39 (s, 3H), 2.23 (t, *J* = 12.3 Hz, 2H), 2.04 (dt, *J* = 14.3, 7.2 Hz, 2H), 1.72 (d, *J* = 13.4 Hz, 2H).MS (ESI) calculated for (C_34_H_33_ClN_8_O_3_) [M + 1]^+^, 637; found, 637.

*Synthesis of 4-{[4-(benzyloxy)-3-chlorophenyl]amino}-2-{[5-methoxy-1*′*-methyl-1 -(prop-2-enoyl)-1,2-dihydrospiro[indole-3,4*′*-piperidine]-6-yl]amino}pyrimidine-5-carbonitrile (* ***A3*** *).* A white solid (TFA salt, 23.1 mg, 38% yield). ^1^H NMR (300 MHz, DMSO-*d*_6_) δ 8.41 (s, 1H), 8.25 (s, 1H), 7.70 (d, *J* = 2.6 Hz, 1H), 7.54 – 7.24 (m, 6H), 6.98 (d, *J* = 9.3 Hz, 1H), 6.92 (s, 1H), 6.72 (dd, J = 16.6, 10.3 Hz, 1H), 6.25 (d, *J* = 16.6 Hz, 1H), 5.81 (d, *J* = 10.4 Hz, 1H), 5.13 (s, 2H), 4.17 (s, 2H), 3.76 (s, 3H), 3.43 (d, *J* = 12.7 Hz, 2H), 3.22 – 3.03 (m, 2H), 2.81 (s, 3H), 2.19 – 1.97 (m, 2H), 1.85 (d, *J* = 13.9 Hz, 2H).MS (ESI) calculated for (C_35_H_34_ClN_7_O_3_) [M + 1]^+^, 636; found, 636.

*Synthesis of 2-{[5-methoxy-1*′*-methyl-1-(prop-2-enoyl)-1,2-dihydrospiro[indole-3,4*′ *-piperidine]-6-yl]amino}-4-{[1-(pyridin-2-ylmethyl)-1H-pyrazol-4-yl]amino}pyrimidine-5-carbonitrile (* ***A4*** *).* a white solid (14 mg, 32% yield). ^1^H NMR (300 MHz, DMSO-*d*_6_) δ 9.65 (s, 1H), 9.12 (s, 1H), 8.56 – 8.44 (m, 1H), 8.36 (s, 1H), 8.22 (s, 1H), 7.71–7.67 (m, 2H), 7.27 (dd, *J* = 7.4, 4.9 Hz, 1H), 7.04 (s, 1H), 6.77 (dd, *J* = 16.5, 10.2 Hz, 1H), 6.22 (dd, *J* = 16.5, 2.3 Hz, 1H), 5.75 (dd, *J* = 10.2, 2.3 Hz, 1H), 5.16 (s, 2H), 3.98 (s, 2H), 3.73 (s, 3H), 2.81 – 2.57 (m, 2H), 2.20 (s, 3H), 2.07 – 1.76 (m, 4H), 1.48 (d, *J* = 11.7 Hz, 2H). MS (ESI) calculated for (C_31_H_32_N_10_O_2_) [M + 1]^+^, 577; found, 577.

*Synthesis of (2E)-4-{6′-[(5-cyano-4-{[4-(pyridin-2-ylmethoxy)phenyl]amino} pyrimidin-2-yl)amino]-5′-methoxyspiro[cyclopropane-1,3′-indole]-1′-yl}-1-(dimethylamino)-4-oxobut-2-en-2-yl (* ***B1*** *).* To a solution of **15a** (40 mg, 0.08 mmol) and *N,N*-diisopropylethylamine (31.53 mg, 0.24 mmol) in dry tetrahydrofuran (2 ml) was added a solution of *(2E)-4-bromobut-2-enoyl chloride* (14.83 mg, 0.08 mmol) in dry tetrahydrofuran (0.5 ml) at 0 °C. The resulting solution was stirred for 1 h at 0 °C. This was followed by the addition of a solution of dimethylamine in tetrahydrofuran (2 ml, 2 M). The resulting solution was allowed to react, with stirring, for an additional 18 h at room temperature. The reaction mixture was diluted with saturated sodium bicarbonate solution (30 ml) and extracted with ethyl acetate (50 ml × 3). The combined organic phase was washed with saturated sodium chloride solution (50 ml × 3), then dried over anhydrous magnesium sulfate, filtered and the filtrate was concentrated under reduced pressure. The residue was purified with Prep-HPLC [Column: Xbridge RP18, 5 um, 19 × 150 mm; mobile phase: water (0.05% ammonium bicarbonate) and acetonitrile (35% acetonitrile up to 65% in 12 min); Detector, UV 220 and 254 nm] to afford **B1** as an off-white solid(1.3 mg, 3% yield). ^1^H NMR (300 MHz, DMSO-*d_6_*) δ 8.97 (s, 1H), 8.81 (s, 1H), 8.56 (d, *J* = 4.2 Hz, 1H), 8.48–8.44 (m, 2H), 7.81 (dd, *J*_1_= 7.8 Hz, *J_2_* = 1.8 Hz), 7.53–7.42 (m, 3H), 7.33–7.31 (m, 1H), 6.98 (d, *J* = 8.7 Hz, 2H), 6.70–6.65 (m, 1H), 6.51 (s, 1H), 6.32 (dd, *J_1_* = 17.1 Hz, *J* = 2.1 Hz, 1H), 5.14 (s, 2H), 4.09 (s, 2H), 3.75 (s, 3H), 2.16 (s, 6H), 1.09 (s, 4H).MS (ESI) calc’d for (C_34_H_34_N_8_O_3_) [M + 1]^+^, 603; found, 603.

Compound B**2-B7** was prepared in analogy to **B1**.

*Synthesis of (2E)-4-{6*′*-[(4-{[3-chloro-4-(pyridin-2-ylmethoxy)phenyl]amino}-5- cyanopyrimidin-2-yl)amino]-5*′*-methoxyspiro[cyclopropane-1,3*′*-indole]-1*′*-yl}-1-(dimethylamino)-4-oxobut-2-en-2-yl (* ***B2*** *).* An off-white solid (1.2 mg, 2% yield). ^1^H NMR (300 MHz, DMSO-*d_6_*) δ 8.81 (s, 1H), 8.58–8.56 (m, 2H), 8.51 (s, 1H), 7.87–7.52 (m, 5H), 7.35–7.31 (m, 1H), 7.16–7.13 (m, 1H), 6.73–6.62 (m, 1H), 6.51 (s, 1H), 6.41–6.31 (m, 1H), 5.22 (s, 2H), 4.12 (s, 2H), 3.76 (s, 3H), 2.16 (s, 6H), 1.08 (s, 4H).MS (ESI) calc’d for (C_34_H_33_ClN_8_O_3_) [M + 1]^+^, 637; found, 637.

*Synthesis of 4-{[4-(benzyloxy)-3-chlorophenyl]amino}-2-({1*′*-[(2E)-4-(dimethylamino) but-2-enoyl]-5*′*-methoxy-1*′*,2*′*-dihydrospiro[cyclopropane-1,3*′*-indole]-6*′*-yl}amino)pyrimidine-5-carbonitrile (* ***B3*** *).* An off-white solid(3.8 mg, 6% yield). ^1^H NMR (300 MHz, DMSO-*d_6_*) δ 8.80 (s, 1H), 8.60 (s, 1H), 8.51 (s, 1H), 7.80 (s, 1H), 7.53–7.37 (m, 7H), 7.14–7.12 (m, 1H), 6.71–6.61 (m, 1H), 6.52 (s, 1H), 6.35–6.27 (m, 1H), 5.16 (s, 2H), 4.11 (s, 2H), 3.76 (s, 3H), 2.15 (s, 6H), 1.12–1.08 (m, 4H).MS (ESI) calc’d for (C_35_H_34_ClN_7_O_3_) [M + 1]^+^, 636; found, 636.

*Synthesis of 3-{6*′*-[(5-cyano-4-{[4-(pyridin-2-ylmethoxy)phenyl]amino}pyrimidin -2-yl) amino]-5*′*-methoxyspiro [cyclopropane-1, 3*′*-indole]-1*′*-yl}-3-oxoprop-1-en-2-yl (* ***B4*** *).* An off-white solid (21.9 mg, 35% yield). ^1^H NMR (300 MHz, DMSO-*d_6_*) δ 8.84 (s, 1H), 8.78 (s, 1H), 8.56 (d, *J* = 4.2 Hz, 1H), 8.48–8.44 (m, 2H), 7.83 (dd, *J*_1_= 7.8 Hz, *J_2_* = 1.8 Hz), 7.77–7.46 (m, 3H), 7.32–7.28 (m, 1H), 6.98 (d, *J* = 8.7 Hz, 1H), 6.54–6.45 (m, 2H), 6.19 (dd, *J_1_* = 17.1 Hz, *J* = 2.1 Hz, 1H), 5.62 (dd, *J_1_* = 10.5 Hz, *J* = 2.1 Hz, 1H), 5.14 (s, 2H), 4.10 (s, 2H), 3.75 (s, 3H), 1.09 (s, 4H). MS (ESI) calc’d for (: C_31_H_27_N_7_O_3_) [M + 1]^+^, 546; found, 546.

*Synthesis of 3-{6*′*-[(4-{[3-chloro-4-(pyridin-2-ylmethoxy)phenyl]amino}-5- cyanopyrimidin-2-yl)amino]-5*′*-methoxyspiro[cyclopropane-1,3*′*-indole]-1*′*-yl}-3-oxoprop-1-en-2-yl (* ***B5*** *).* An off-white solid (1.6 mg, 1% yield). ^1^H NMR (300 MHz, DMSO-*d_6_*) δ 8.91 (s, 1H), 8.56 (d, *J* = 4.2 Hz, 2H), 8.51 (s, 1H), 7.85–7.82 (m, 2H), 7.56–7.51 (m, 2H), 7.34–7.28 (m, 1H), 7.16–7.12 (m, 1H), 6.56–6.51 (m, 2H), 6.19 (d, *J* = 10.5 Hz, 1H), 5.62 (d, *J* = 10.5 Hz, 1H), 5.22 (s, 2H), 4.12 (s, 2H), 3.77 (s, 3H), 1.10 (d, *J* = 6.0 Hz, 4H). MS (ESI) calc’d for (C_31_H_26_ClN_7_O_3_) [M + 1]^+^, 580; found, 580.

*Synthesis of (2E)-4-{6*′*-[(5-cyano-4-{[1-(pyridin-2-ylmethyl)pyrazol-4-yl]amino} pyrimidin-2-yl)amino]-5*′*-methoxyspiro[cyclopropane-1,3*′*-indole]-1*′*-yl}-1-(dimethylamino)-4-oxobut-2-en-2-yl trifluoroacetate (* ***B6*** *).* A yellow solid (3.8 mg, 6% yield). ^1^H NMR (300 MHz, DMSO-*d_6_*) δ 9.35 (s, 1H), 9.14 (s, 1H), 8.70 (s, 1H), 8.52 (d, *J* = 8.4 Hz, 1H), 8.44 (s, 1H), 8.03 (s, 1H), 7.78–7.72 (m, 2H), 7.32–7.28 (m, 1H), 7.12–7.09 (m, 1H), 6.76–6.55 (m, 3H), 5.37 (s, 2H), 4.16 (s, 2H), 3.90 (d, *J* = 2.8 Hz, 2H), 3.78 (s, 3H), 2.82 (s, 6H), 1.13 (t, *J* = 6.7 Hz, 4H). MS (ESI) calc’d for: (C_31_H_32_N_10_O_2_) [M + 1]^+^, 577; found, 577.

*Synthesis of 3-{6*′*-[(5-cyano-4-{[1-(pyridin-2-ylmethyl)pyrazol-4-yl]amino} pyrimidin-2-yl)amino]-5*′*-methoxyspiro[cyclopropane-1,3*′*-indole]-1*′*-yl}-3-oxoprop-1-en-2-yl (* ***B7*** *).* An off-white solid (12 mg, 27% yield). ^1^H NMR (300 MHz, DMSO-*d_6_*) δ 9.74 (s, 1H), 9.32 (s, 1H), 8.80 (s, 1H), 8.55 (d, *J* = 8.4 Hz, 2H), 8.11 (s, 1H), 7.80–7.77 (m, 2H), 7.34–7.30 (m, 1H), 7.06 (d, *J* = 8.4 Hz, 1H), 6.63–6.58 (m, 2H), 6.24 (d, *J* = 17.7 Hz, 1H), 5.72 (d, *J* = 8.4 Hz, 1H), 5.42 (s, 2H), 4.17 (s, 2H), 3.78 (s, 3H), 1.13 (t, *J* = 8.7 Hz, 4H). MS (ESI) calc’d for: (C_28_H_25_N_9_O_2_) [M + 1]^+^, 520; found, 520.

*Synthesis of (2E)-4-{6-[(5-cyano-4-{[4-(pyridin-2-ylmethoxy)phenyl]amino} pyrimidin-2-yl)amino]-7-methoxyspiro[1,4-benzoxazine-2,1′-cyclopropane]-4-yl}-1-(dimethylamino)-4-oxobut-2-en-2-yl (* ***C1*** *).* To a solution of **21a** (100 mg, 0.20 mmol) and triethylamine (298.8 mg, 2.95 mmol) in dichloromethane (10 ml) was added a solution of *(2E)*-4-(dimethylamino)but-2-enoyl chloride hydrochloride (360.9 mg, 1.96 mmol) in dichrolomethane (3 ml) at 0 °C. The resulting solution was stirred for 2 h at 40 °C. The reaction was quenched with saturated sodium bicarbonate solution (30 ml) and extracted with dichloromethane (30 ml × 3). The combined organic phase was washed with brine (50 ml × 3), then dried over anhydrous magnesium sulfate, filtered and the filtrate was concentrated under reduced pressure. The residue was purified by preparative HPLC [Column: X Bridge C18, 19*150 mm, 5 um; Mobile Phase A:Water with 10 mM ammonium bicarbonate, Mobile Phase B: acetonitrile; Flow rate: 20 ml/min; Gradient: 20% B to 45% B in 10.0 min; 254 nm] to afford **C1** as an off-white solid (8.8 mg, 7% yield). ^1^H NMR (300 MHz, DMSO-*d_6_*) δ 9.25 (br, 1H), 8.73–8.61 (m, 1H), 8.56 (d, *J* = 4.2 Hz, 1H), 8.40 (s, 1H), 7.83 (dd, *J*_1_= 7.8 Hz, *J_2_* = 1.8 Hz, 1H), 7.55–7.38 (m, 5H), 6.90–6.50 (m, 5H), 5.13 (s, 2H), 3.89 (s, 2H), 3.72 (s, 3H), 2.90 (br, 2H), 2.02 (br, 6H), 0.96–0.94 (m, 2H), 0.78–0.76(m, 2H). MS (ESI) calc’d for (C_34_H_34_N_8_O_4_) [M + 1]^+^, 619; found, 619.

Compound C**2-C7** was prepared in analogy to **C1**.

*Synthesis of (2E)-4-{6-[(4-{[3-chloro-4-(pyridin-2-ylmethoxy)phenyl]amino}-5- cyanopyrimidin-2-yl)amino]-7-methoxyspiro[1,4-benzoxazine-2,1*′*-cyclopropane]-4-yl}-1-(dimethylamino)-4-oxobut-2-en-2-yl(* ***C2*** *).* An off-white solid (15.3 mg, 25% yield). ^1^H NMR (300 MHz, DMSO-*d_6_*) δ 9.04 (br, 1H), 8.56 (s, 1H), 8.47 (s, 1H), 8.37 (s, 1H), 7.86–7.80 (m, 1H), 7.65–7.55 (m, 3H), 7.41–7.28 (m, 2H), 6.98 (d, *J* = 3.2 Hz, 1H), 6.62–6.55 (m, 2H), 6.45–6.37 (m, 1H), 5.20 (s, 2H), 3.88 (s, 2H), 3.72 (s, 3H), 2.99–2.87 (m, 2H), 2.04 (s, 6H), 0.96–0.94 (m, 2H), 0.78–0.76(m, 2H). MS (ESI) calc’d for (C_34_H_33_ClN_8_O_4_) [M + 1]^+^, 653; found, 653.

*Synthesis of (2E)-4-{6-[(4-{[4-(benzyloxy)-3-chlorophenyl]amino}-5-cyanopyrimidin -2-yl)amino]-7-methoxyspiro[1,4-benzoxazine-2,1*′*-cyclopropane]-4-yl}-1-(dimethylamino)-4-oxobut-2-en-2-yl (* ***C3*** *).*an off-white solid (14.5 mg, 15% yield). ^1^H NMR (300 MHz, DMSO-*d_6_*) δ 9.02 (br, 1H), 8.46 (s, 1H), 8.37 (s, 1H), 7.64 (s, 2H), 7.47–7.32 (m, 6H), 7.02 (d, *J* = 3.2 Hz, 1H), 6.63–6.57 (m, 2H), 6.44–6.39 (m, 1H), 5.14 (s, 2H), 3.88 (s, 2H), 3.72 (s, 3H), 2.93–2.87 (m, 2H), 2.05 (s, 6H), 0.96–0.94 (m, 2H), 0.78–0.76(m, 2H). MS (ESI) calc’d for (C_35_H_34_ClN_7_O_4_) [M + 1]^+^, 652; found, 652.

*Synthesis of (2E)-4-{6-[(5-cyano-4-{[1-(pyridin-2-ylmethyl)pyrazol-4-yl]amino} pyrimidin-2-yl)amino]-7-methoxyspiro[1,4-benzoxazine-2,1*′*-cyclopropane]-4-yl}-1-(dimethylamino)-4-oxobut-2-en-2-yl (* ***C4*** *).* An off-white solid (23.2 mg, 19% yield). H NMR (300 MHz, DMSO-*d_6_*) δ 9.41 (s, 1H), 8.76 (s, 1H), 8.50 (d, *J* = 3.2 Hz, 1H), 8.32 (s, 1H), 7.90–7.80(m, 1H), 7.70–7.53 (m, 3H), 7.28–7.24 (m, 1H), 6.90 (d, *J* = 3.2 Hz, 1H), 6.70–6.47 (m, 3H), 5.19 (s, 2H), 3.85 (s, 2H), 3.68 (s, 3H), 2.91 (br, 2H), 2.06 (s, 6H), 0.96–0.94 (m, 2H), 0.78–0.76(m, 2H). MS (ESI) calc’d for (C_31_H_32_N_10_O_3_) [M + 1]^+^, 593; found, 593.

### 3.2. Biological activity evaluation

#### 3.2.1. EGFR-wt/HER2 protein kinase assay

The inhibitory profile of 16 compounds was determined using two protein kinases. The compounds were tested at a concentration of 5 × 1 0^−7 ^M, in singlicate. The compounds were solved and diluted to 5 × 10^−5 ^M stock solutions in 100% DMSO according to the customer-filled CSF. 10 μL of each of the stock solutions were transferred into column 3–5 of a 96-well, Column 1 and 2 of the plate were filled with 10 μL100% DMSO. The plate is in the following referred to as **“copy plate”**. In the process, 90 μL H_2_O were added to each well of the copy plate. To minimize potential compound precipitation, the H_2_O was added to each well only a few minutes before the transfer of the compound solutions into the assay plates. The plate was shaken thoroughly, resulting in a **“compound dilution plate”** with a compound concentration of 5 × 10^−6 ^M/10% DMSO. This plate was used for the transfer of 5 μL compound solution into the **assay plates**. The final volume of the assay was 50 μL. All compounds were tested at 5 × 1 0^−7 ^M in singlicate. The final DMSO concentration in the reaction cocktails was 1% in all cases. The compound dilution plate was disposed.

A radiometric protein kinase assay (33PanQinase® Activity Assay) was used for measuring the kinase activity of the two protein kinases. All kinase assays were performed in 96-well FlashPlatesTM from PerkinElmer (Boston, MA, USA) in a 50 μL reaction volume. The reaction cocktail was pipetted in 4 steps in the following order: 20 μL of assay buffer, 5 μL of ATP solution (in H2O), 5 μL of test compound (in 10% DMSO), 20 μL enzyme/subtrate mix. The assay for both protein kinases contained 70 mM HEPES-NaOH pH 7.5, 3 mM MgCl_2_, 3 mM MnCl_2_, 3 μM Na-orthovanadate, 1.2 mM DTT, ATP. The assay for ERBB2 additionally contained 50 μg/mLPEG20000. The reaction cocktails were incubated at 30 °C for 60 min. The reaction was stopped with 50 μL of 2% (v/v) H_3_PO_4_, plates were aspirated and washed two times with 200 μL 0.9% (w/v) NaCl. Incorporation of 33Pi was determined with a microplate scintillation counter (Microbeta, Wallac). All assays were performed with a BeckmanCoulter/SAGIAN™Core System. The median value of the counts in column 1 (*n* = 8) of each assay plate was defined as **“low control”**. This value reflects unspecific binding of radioactivity to the plate in the absence of a protein kinase but in the presence of the substrate. The median value of the counts in column 2 of each assay plate (*n* = 8) was taken as the **“high control”**. As part of the data evaluation the low control value from a particular plate was subtracted from the high control value as well as from all 80 “compound values” of the corresponding plate. The residual activity (in %) for each well of a particular plate was calculated by using the following formula:

Res. Activity (%) = 100 × [(cpm of compound – low control)/(high control − low control)]

#### 3.2.2. Seven EGFR mutants protein kinase assay

The compounds were serially diluted in semi-log steps with 100% DMSO in a 96 well microtiter plate. The concentration range of this serial dilution was 1 × 10^−3 ^M to 3 × 10^−8 ^M. Directly before use the test compounds were further diluted 1:10 with water, resulting in a concentration range from 1 × 10^−4 ^M to 3 × 1 0^−9 ^M in 10% DMSO. For the assays, 5 μL from each concentration were transferred into the assay. The final volume of the assay was 50 μL. The compounds were tested at 10 final assay concentrations in the range from 1 × 10^−5 ^M to 3 × 10^−10 ^M. The final DMSO concentration in the reaction cocktails was 1% in all cases.

A radiometric protein kinase assay (33PanQinase^®^ Activity Assay) was used for measuring the kinase activity of the 9 protein kinases. All kinase assays were performed in 96-well FlashPlatesTM from Perkin Elmer (Boston, MA, USA) in a 50 μL reaction volume. The reaction cocktail was pipetted in 4 steps in the following order: 10 μL of non-radioactive ATP solution (in H_2_O), 25 μL of assay buffer/[γ-33P]-ATP mixture, 5 μL of test sample in 10% DMSO, 10 μL of enzyme/substrate mixture. The assay for all protein kinases contained 70 mM HEPES-NaOH pH 7.5, 3 mM MgCl_2_, 3 mM MnCl_2_, 3 μM Na-orthovanadate, 1.2 mM DTT, ATP, [γ-33P]-ATP, protein kinase, and substrate.The reaction cocktails were incubated at 30 °C for 60 min. The reaction was stopped with 50 μL of 2% (v/v) H_3_PO_4_, plates were aspirated and washed two times with 200 μL 0.9% (w/v) NaCl. Incorporation of 33Pi (counting of “cpm”) was determined with a microplate scintillation counter (Microbeta, Wallac). All assays were performed with a BeckmanCoulter Biomek 2000/SL robotic system.

For each kinase, the median value of the cpm of three wells with complete reaction cocktails, but without kinase, was defined as “low control” (*n* = 3). This value reflects unspecific binding of radioactivity to the plate in the absence of protein kinase but in the presence of the substrate. Additionally, for each kinase the median value of the cpm of three other wells with the complete reaction cocktail, but without any compound, was taken as the “high control”, i.e. full activity in the absence of any inhibitor (*n* = 3). The difference between high and low control was taken as 100% activity for each kinase. As part of the data evaluation the low control value of each kinase was subtracted from the high control value as well as from their corresponding “compound values”. The residual activity (in %) for each compound well was calculated by using the following formula:

Res. Activity (%) = 100 × [(cpm of compound – low control)/(high control – low control)]

Since 10 distinct concentrations of each compound were tested against each kinase, the evaluation of the raw data resulted in 10 values for residual activities per kinase. Based on each 10 corresponding residual activities, IC50 values were calculated using Prism 5.04 for Windows. The mathematical model used was “Sigmoidal response (variable slope)” with parameters “top” fixed at 100% and “bottom” at 0%. The fitting method used was a least-squares fit.

### 3.3. Molecular docking

The docking was performed with MOE (version 2019)^22^. The crystal structures of EGFR mutations (T790M/L858R and T790M) with neratinib (PDB code: 3W2Q and 2JIV) were downloaded from Protein Data Bank (PDB: http://www.rcsb.org/). Only A chain of EGFR and neratinib were kept, and then the complexes were prepared by using MOE QuickPrep module in MOE (Molecular Operating Environment, version 2019.01) with default parameters. The compounds A1 and A2 were optimized based on MMFF94X force field. The MOE covalent docking was employed to predict the covalent binding modes of EGFR protein with A1, A2 and neratinib. In our covalent docking study, this method relies on a reaction/transformation placement methodology to match the reactive groups (ethylene bond) on the A1, A2 and neratinib and the cysteine residue followed by the formation of the covalent bond between them. Covalent ligands were modeled and minimized in the prereaction form generated with the Builder panel module. The default postprocessing protocol using minimization by the Rigid Receptor refinement scheme and rescoring by the GBVI/WSA dG scoring function was used to estimate the binding free energy (Kcal/mol) of docked pose and EGFR protein. A total of 30 covalent docking poses for each compound were ranked by the estimated binding free energy.

## Supplementary Material

Supplemental Material
