# Supplementary material for: Discovery of aminopyridine-containing spiro derivatives as EGFR mutations inhibitors
Source: J Enzyme Inhib Med Chem. 2019 Jul 9;34(1):1233–46. doi: 10.1080/14756366.2019.1634704 (PMC6691816; doi:10.1080/14756366.2019.1634704)
Supplement: Supplemental Material [file IENZ_A_1634704_SM7194.doc]

# Discovery of aminopyridine-containing spiro derivatives as EGFR mutations inhibitors

Supplementary information


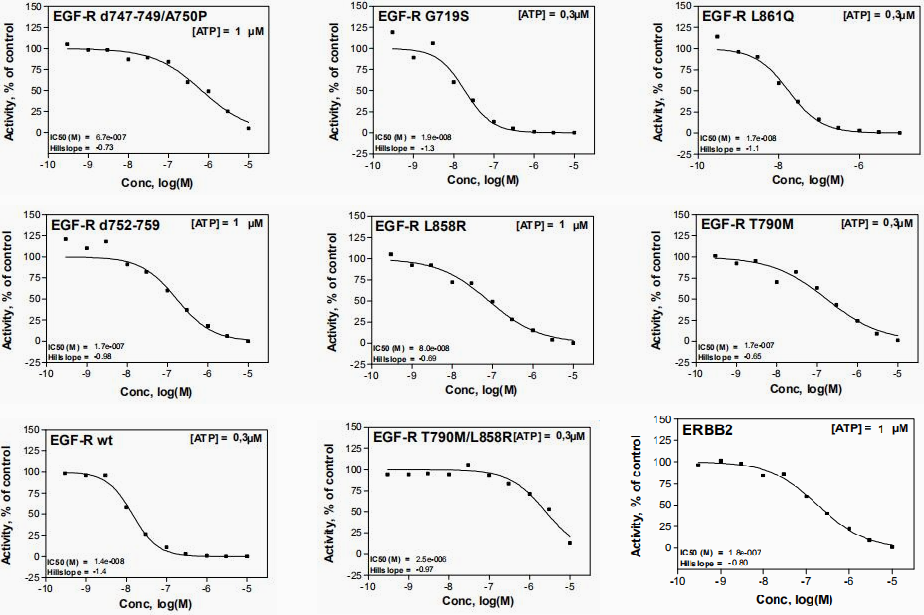


**Additional file 1: Figure S1**. IC_50_ curves of neratinib in 9 protein kinase assays


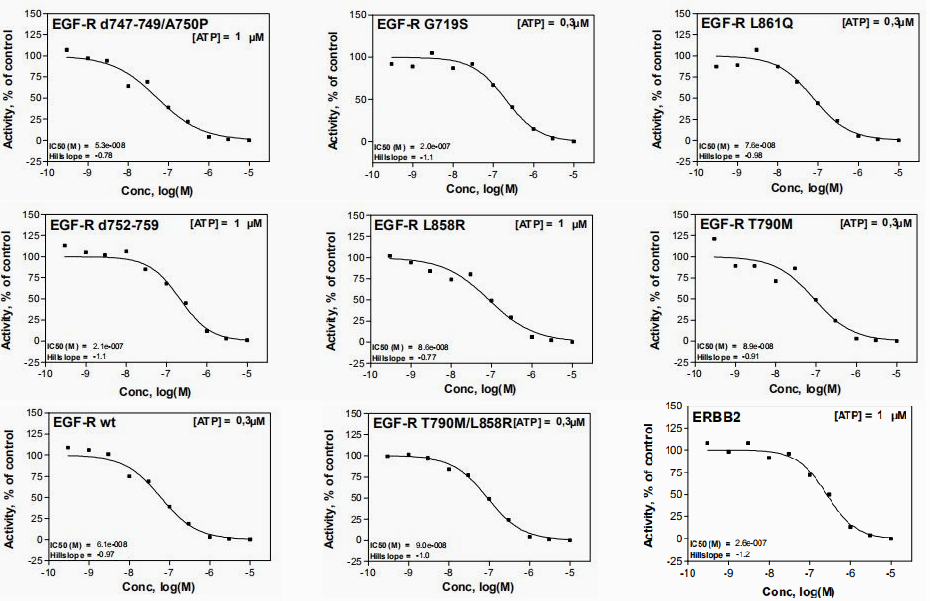


**Additional file 2: Figure S2**. IC_50_ curves of A1 in 9 protein kinase assays


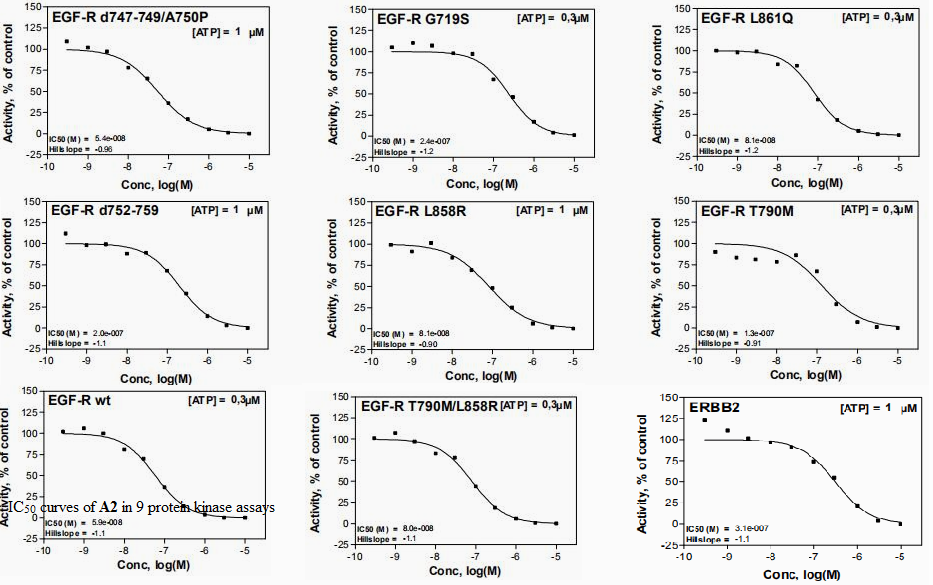


**Additional file 3: Figure S3**. IC_50_ curves of A2 in 9 protein kinase assays
